# A three-dimensional phase diagram of growth-induced surface instabilities

**DOI:** 10.1038/srep08887

**Published:** 2015-03-09

**Authors:** Qiming Wang, Xuanhe Zhao

**Affiliations:** 1 Soft Active Materials Laboratory, Department of Mechanical Engineering, Massachusetts Institute of Technology, Cambridge, Massachusetts 02139, USA; 2 Department of Mechanical Engineering and Materials Science, Duke University, Durham, North Carolina 27708, USA; 3 Department of Civil and Environmental Engineering, Massachusetts Institute of Technology, Cambridge, MA 02139, USA

## Abstract

A variety of fascinating morphological patterns arise on surfaces of growing, developing or aging tissues, organs and microorganism colonies. These patterns can be classified into creases, wrinkles, folds, period-doubles, ridges and delaminated-buckles according to their distinctive topographical characteristics. One universal mechanism for the pattern formation has been long believed to be the mismatch strains between biological layers with different expanding or shrinking rates, which induce mechanical instabilities. However, a general model that accounts for the formation and evolution of these various surface-instability patterns still does not exist. Here, we take biological structures at their current states as thermodynamic systems, treat each instability pattern as a thermodynamic phase, and construct a unified phase diagram that can quantitatively predict various types of growth-induced surface instabilities. We further validate the phase diagram with our experiments on surface instabilities induced by mismatch strains as well as the reported data on growth-induced instabilities in various biological systems. The predicted wavelengths and amplitudes of various instability patterns match well with our experimental data. It is expected that the unified phase diagram will not only advance the understanding of biological morphogenesis, but also significantly facilitate the design of new materials and structures by rationally harnessing surface instabilities.

Numerous intriguing morphologies and phenomena on surfaces of growing animals, plants and microorganism colonies have fascinated artists and scientists for decades^1^,^2^. Abundant examples (Fig. 1A) can be found in various types of living creatures across multiple size scales, such as wrinkles on skins of mammalians, plants and fruits^3^,^4^,^5^,^6^,^7^,^8^, undulations in developing biofilms^9^,^10^,^11^, grooves on the cerebral cortex^12^,^13^,^14^,^15^, mucosal villi and folds of airways, esophagi and guts^16^,^17^,^18^,^19^,^20^,^21^,^22^, buckled tumor surfaces^23^,^24^, epithelial cell delamination due to tissue crowding^25^,^26^, and crumpled membranes of blood cells^27^. Although these biological patterns may be results of complex genetic, biological and biochemical processes, recent studies have suggested that growth-induced mechanical forces regulate the formation and evolution of biological patterns^2^,^16^,^18^,^28^,^29^,^30^. Biological structures usually consist of multiple layers with strikingly different biochemical compositions and mechanical properties; for example, epidermis on the dermis or hypodermis of mammalian skins^3^,^4^,^5^, the epidermis on the ground tissue of plant skins^6^,^7^,^8^, biofilms on culture gels^9^,^10^,^11^, the grey matter on the white matter of cerebral cortexes^12^,^13^, the mucosa on the muscle layer of airways, esophagi and guts^16^,^17^,^18^,^19^,^20^,^21^,^22^, outer proliferative cells on the inner necrotic core of a tumor^23^, epithelial cell monolayer on the underlying tissue^25^,^26^, membranes on the cytoskeleton of blood cells^27^. During growth, development or aging, different layers of biological structures usually have different expanding or shrinking rates, thus resulting in mismatch strains between the biological layers. The surface topographical patterns have long been believed to be results of mismatch-induced compressive strains in the skin layers which have higher growth rates or lower shrinkage rates than the underlying biological layers^14^,^15^,^16^,^18^,^31^. Once the mismatch compressive strain rises to critical values, the initially flat surface of the film becomes unstable and bifurcate into different types of corrugated patterns (Fig. 1B), including (i) *wrinkle* — the film undulates sinusoidally but remains locally smooth (e.g., the pumpkin skin in Figs 1Ai and 1Bi)^6^, (ii) c*rease* — the surface of the film folds into dispersed regions of self-contacts with sharp tips (e.g., the cerebral cortex in Figs 1Aii and 1Bii)^12^,^32^, and (iii) *delaminated-buckle* — the film delaminates from the substrate to form buckled regions (e.g., the biofilm in Figs 1Aiii and 1Biii)^10^. As the mismatch strain further increases, the wrinkles may further bifurcate into more complicated surface patterns, including (iv) *fold* – some valleys of the wrinkle fold into self-contacts with sharp tips (e.g., the dog skin in Figs 1Aiv and 1Biv)^33^, (v) *period-double* — the sinusoidal wrinkle transits into a pattern with twice of the wavelength (Fig. 1Bv), and (vi) *ridge* — the wrinkle drastically increases its amplitude but decreases its wavelength, forming a high-aspect-ratio pattern that ceases to follow sinusoidal shape (Fig. 1Bvi). These instability patterns with distinctive topographical characteristics have been only studied and identified separately in different biological systems under varied physical and biological conditions^6^,^10^,^16^,^18^,^25^. However, a general model that can quantitatively predict the formation and evolution of various types of surface-instability patterns still does not exist; primarily because existing theories such as linear stability analysis cannot systematically analyze all modes of instabilities^12^, and existing experiments did not systematically vary the mechanical properties of film-substrate systems.

Here, we take biological film-substrate structures at their current states as thermodynamic systems, and regard each mode of surface-instability pattern as a thermodynamic phase. By systematically varying mechanical properties of the structure including moduli, adhesion energy and mismatch strain of the film and substrate, we calculate the initiation and evolution of various modes of growth-induced surface instabilities. We then compare potential energies of different instability patterns, and construct a quantitative phase diagram that accounts for all instability patterns discussed above, by assuming the current pattern seeks the lowest potential energy among all possible configurations. To validate the phase diagram, we impose different mismatch strains in polymeric film-substrate structures with systematically varied rigidity and adhesion energy to induce various modes of instability patterns. The resultant patterns indeed follow the phase diagram quantitatively. We further find that the phase diagram agrees well with reported data on growth-induced surface instabilities from a number of previous studies. It is expected that the phase diagram will not only advance the understanding of biological morphogenesis, but also significantly facilitate the design of new structures with innovative surfaces or interfaces for disease therapy^22^,^24^, active cell culture^34^, biofouling management^35^, tunable superhydrophobicity^36^ and flexible electronics^37^,^38^.

## Results

### A three-dimensional phase diagram

**Figure 1:**
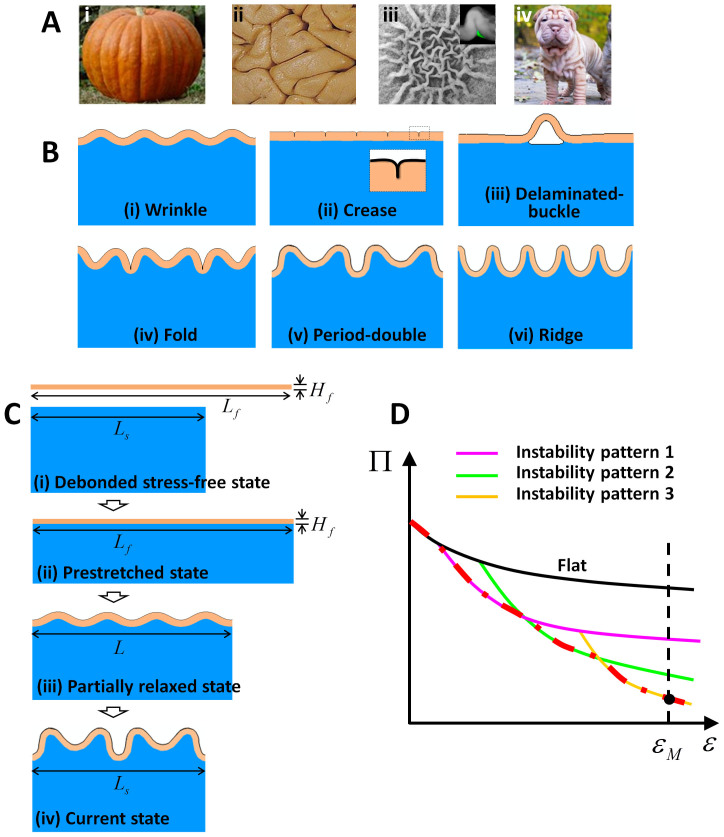
Illustrations of examples, schematics and potential energies of various growth-induced surface instabilities. (A) Examples of growth-induced surface instabilities on (i) the pumpkin skin, (ii) the cerebral cortex, (iii) the biofilm and (iv) the dog skin. (B) Schematics of growth-induced surface instabilities: (i) wrinkle, (ii) crease, (iii) delaminated-buckle, (iv) fold, (v) period-double and (vi) ridge. (C) One example pathway to induce the mismatch strain in the film-substrate structure: (i) The film and substrate is first assumed to be detached from each other to form a stress-free state; (ii) the detached stress-free substrate is then pre-stretched by a ratio of *L_f_*/*L_s_* and adhered to the film; (iii) relaxed to length *L*; and (iv) eventually relaxed to length *L_s_* at the current state. Other pathways to induce mismatch strains are illustrated in Supplementary Figs S5 and S7. (D) Evolution of potential energy of the film-substrate structure with increasing mismatch strain following the pathway in (C). The red dash line denotes the surface patterns with the minimum potential energy. The potential energy of the film-substrate structure with mismatch strain *ε_M_* is denoted by the black solid circle. Image (Ai) is reprinted with permission from Yin, et al., Proc. Natl. Acad. Sci. U.S.A., 105, 49 (2008). Copyright 2008, National Academy of Sciences, USA. Image (Aii) is reprinted from Bradbury, PLOS Biol., 3, 3 (2005) under Open-Access License. Image (Aiii) is reprinted with permission from Asally, et al., Proc. Natl. Acad. Sci. U.S.A., 109, 46 (2012). Copyright 2012, National Academy of Sciences, USA. Image (Aiv) is reprinted with permission from Alison Ruhe.

While the development of instability patterns in biological structures may involve complicated biological processes, determining the instability patterns at current states can be solved as mechanics problems^2^,^16^,^18^,^28^,^29^,^30^. To focus on essential physical features, we simplify the layered biological structures at the current states as a homogeneous film adhered on a homogeneous underlying substrate, both undergoing plane-strain deformation (Fig. 1C). To account for large deformation, both the film and the substrate are taken as incompressible neo-Hookean materials with shear modulus *μ_f_* and *μ_s_*, respectively. If the film and the substrate at the current state are detached from each other, they will have lengths *L_f_* and *L_s_* and thicknesses *H_f_* and *H_s_*, respectively (Fig. 1Ci). We define the mismatch strain between the film and the substrate at current state as *ε_M_* = (*L_f_* − *L_s_*)/*L_f_*. Since film thickness *H_f_* is much smaller than all the other dimensions (i.e., *L_f_*, *L_s_* and *H_s_*) in the system, it is the only relevant length scale for analyzing the instability patterns. We further define the adhesion energy between the film and the substrate, Γ, as the work required to detach the film from a unit area of the substrate in the stress-free state.

Within the time scale of determining instability patterns, we take the film-substrate structure as a thermodynamic system, and assume the current surface-instability pattern always seeks the lowest potential energy among all possible configurations (Fig. 1D), *i.e.*, following the Maxwell stability criterion^39^,^40^,^41^,^42^. The potential energy per unit width of the film-substrate system under plane-strain deformation can be expressed as^39^
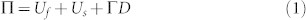
where *U_f_* and *U_s_* are strain energies per unit width of the film and substrate, respectively, and *D* is the current delaminated length of the substrate measured in the stress-free state (Fig. 1Ci). This simplified model involves five physical parameters that determine the instability patterns: *μ_f_*, *μ_s_*, *H_f_*, Γ and *ε_M_*. By dimensional argument, they can be normalized into three dimensionless parameters: modulus ratio *μ_f_*/*μ_s_*, normalized adhesion energy Γ/(*μ_s_H_f_*) and mismatch strain *ε_M_*. The types of instability patterns will be solely determined by the three dimensionless parameters, and therefore governed by a three-dimensional phase diagram. It should be noted that biological structures can take different paths to induce mismatch strains such as expansion of films or shrink of substrates (see *e.g.*, Supplementary Figs S5 and S7); however, structures with the same set of *μ_f_*/*μ_s_*, Γ/(*μ_s_H_f_*) and *ε_M_* should reach the same type of instability pattern at the current state, given the Maxwell stability criterion is followed.

**Figure 2:**
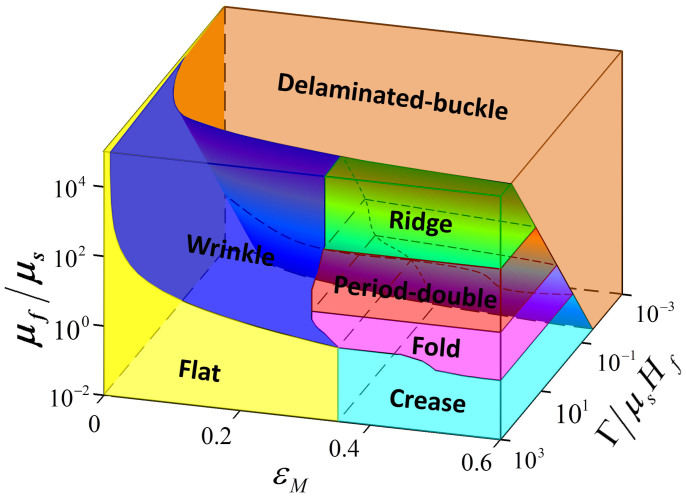
A calculated three-dimensional phase diagram of various surface instability patterns induced by mismatch strains. The instability pattern is determined by three non-dimensional parameters: mismatch strain *ε_M_*, modulus ratio *μ_f_*/*μ_s_* and normalized adhesion energy Γ/(*μ_s_H_f_*).

Next, we discuss the process to quantitatively construct the phase diagram. A plane-strain finite element model is developed to calculate the formation of instability patterns (Methods and SI). To induce mismatch strains in the model, we assume the detached stress-free substrate in Fig. 1Ci is pre-stretched by a ratio of *L_f_*/*L_s_*, adhered to the film (Fig. 1Cii), and then relaxed to length *L* (Fig. 1Ciii), during which all deformation occurs in plane-strain condition. The overall compressive strain in the film is defined as *ε* = (*L_f_* − *L*)/*L_f_* (Fig. 1Ciii). As *ε* increases to critical values, patterns of surface instabilities can initiate and transit into others (Fig. 1D). Force perturbations and mesh defects have been introduced into the model as fluctuations to facilitate the system to seek minimum-potential energy states (Fig. 1D). When the substrate is fully relaxed (*i.e.*, *L* = *L_s_* and *ε* = *ε_M_*, shown as the black solid circle on Fig. 1D), the resultant pattern is the instability pattern of the film-substrate system with mismatch strain *ε_M_*, which represents a point of one phase in the phase diagram (Fig. 2). The boundaries between regions of different phases give the phase boundaries on the phase diagram. We can also determine the phase boundaries by comparing the potential energies of different patterns with the same set of *μ_f_*/*μ_s_*, Γ/(*μ_s_H_f_*) and *ε_M_*^39^,^43^,^44^, i.e.,
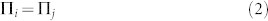
where Π*_i_* and Π*_j_* are the potential energies of two different patterns on film-substrate models with the same properties and dimensions (Fig. 1D). Following this method, we categorize all modes of surface instabilities patterns discussed above into a three-dimensional phase diagram with quantitatively determined phase boundaries (Fig. 2).

**Figure 3:**
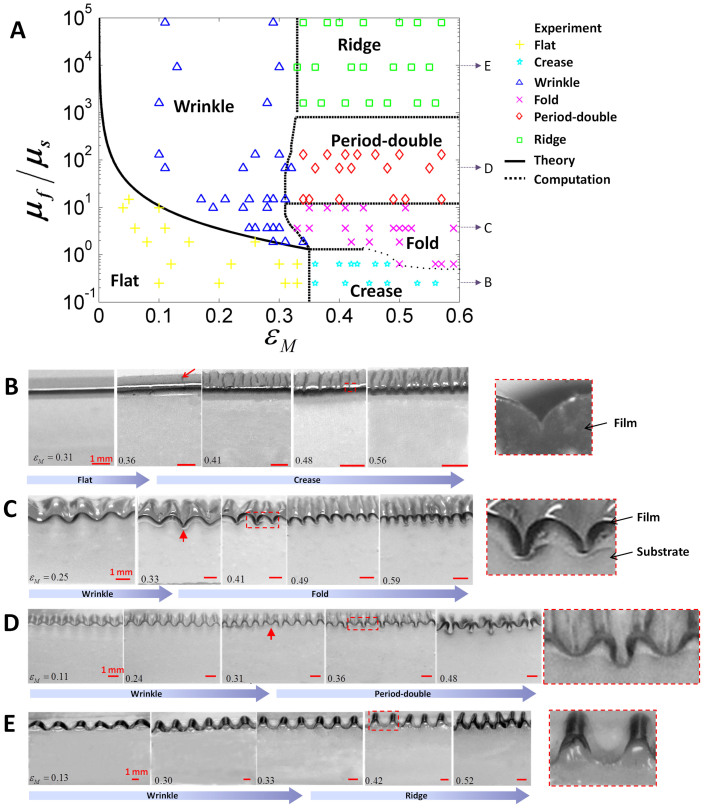
Experimental validation of the phase diagram for instability patterns in film-substrate structures with high adhesion energies. (A) Comparison between experimental data and the phase diagram of surface instability patterns without delamination. Experimental images to show the formation of (B) creases, (C) wrinkles and folds, (D) wrinkles and period-doubles, and (E) wrinkles and ridges in film-substrate structures with different modulus ratios and mismatch strains. The film-substrate modulus ratios are (B) 0.3, (C) 3.64, (D) 67.24 and (E) 9110, respectively.

To understand the phase diagram, we first consider the scenario in which the adhesion between the film and the substrate is so strong that the film does not delaminate from the substrate (*i.e.*, *D* = 0). The instability patterns are thus governed only by *μ_f_*/*μ_s_* and *ε_M_*, giving a two-dimensional phase diagram (*i.e.*, Γ/(*μ_s_H_f_*)→∞ on Figs 2 and 3A). When the mismatch strain *ε_M_* is sufficiently low, the flat film-substrate structure has lower potential energy than any instability pattern. As the mismatch strain increases to critical values, the flat state will transit into either wrinkled or creased state, depending on the modulus ratio. When *μ_f_*/*μ_s_* < 1.3 (*i.e.*, relatively compliant film), the film tends to fold against itself without deforming the substrate to minimize the potential energy of the system. The phase boundary between the flat and creased states, which is calculated by setting Π*_flat_* = Π*_crease_*^12^,^43^, is a vertical line on Figs 2 and 3A,
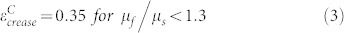
where 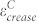 is the critical mismatch strain, at which the structure transits from flat to creased state. It is noted that, for 0.5 < *μ_f_*/*μ_s_* < 1.3, the creases may further develop into folds under larger mismatch strains, *i.e.,* *ε_M_* > 0.45 (Supplementary Fig. S1).

On the other hand, when *μ_f_*/*μ_s_* > 1.3 (*i.e.*, relatively stiff film), the film tends to undulate together with the substrate to minimize the potential energy of the system. The phase boundary between the flat and creased states, which is calculated by setting Π*_flat_* = Π*_wrinkle_* (See SI and Supplementary Fig. S2), can be approximated as a curve on Figs 2 and 3A,
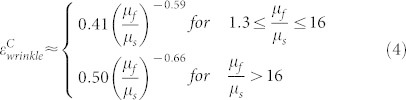
where 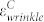 is the critical mismatch strain, at which the structure transits from flat to wrinkle state. Notably, the triple point between flat, creased and wrinkled states in a film-substrate structure with mismatch strain is at *ε_M_* = 0.35 and *μ_f_*/*μ_s_* = 1.3.

As the mismatch strain further increases, the wrinkled film-substrate structure can further bifurcate into more complicated patterns including fold, period-double and ridge, depending on the modulus ratio *μ_f_*/*μ_s_*. Qualitatively, the pattern of fold develops at a lower range of *μ_f_*/*μ_s_* (*i.e.*, relatively compliant films) than period double and ridge, because the fold requires large deformation and self-contact of films. Quantitatively, the calculated phase boundaries between fold, period-double and ridge are approximately horizontal lines at *μ_f_*/*μ_s_* ≈ 12 and *μ_f_*/*μ_s_* ≈ 800, respectively, in the region of 0.3 < *ε_M_* < 0.6 (Figs 2 and 3A). In addition, the calculated phase boundary between wrinkle and fold is in the region of 1.3 < *μ_f_*/*μ_s_* < 12 and 0.3 < *ε_M_* < 0.35 (Figs 2 and 3A, Supplementary Fig. S3); between wrinkle and period double in the region of 12 < *μ_f_*/*μ_s_* ≤ 800 and 0.3 < *ε_M_* < 0.32 (Figs 2 and 3A, Supplementary Figs S4 and S5); and between wrinkle and ridge states in the region of 800 < *μ_f_*/*μ_s_* < 10^6^ and *ε_M_* ≈ 0.33 (Figs 2 and 3A, Supplementary Figs S6 and S7). If the mismatch strain increases beyond 0.6, more complicated patterns will develop, such as period triple^45^,^46^, period quadruples^45^, and co-existence of fold, period double and ridge, which will be not be covered in the current paper. It should be noted, since the Maxwell stability criterion is followed here, the formation of these instability patterns is independent on the processes of inducing mismatch strains. Our finite-element calculations indeed give the same instability patterns, while following different pathways to induce the mismatch strains, such as substrate pre-stretching and relaxing, film swelling and substrate shrinking (Fig. 1C, Supplementary Figs S5 and S7).

Next, we discuss the scenario in which the adhesion between the film and substrate is relatively weak (i.e., relatively low Γ/(*μ_s_H_f_*)) and the film debonds from the substrate to form delaminated-buckles (i.e., *D* > 0). To calculate the potential energy of a delaminated-buckled pattern Π*_buckle_*, we first prescribe various pairs of delaminated length *D* and delaminated-buckle wavelength *λ* in the finite-element model (See Supplementary Figs S8A, S9A and S10A). Minimization of the potential energy of the structure requires^47^
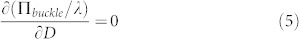

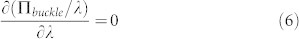


By solving Eqs. 5 and 6, we can obtain a set of *D*, *λ* and Π*_buckle_* for the film-substrate structure, which is assumed to be in the delaminated-buckled state (Supplementary Figs S8B, S9B and S10B). To calculate the phase boundary between the delaminated-buckle and other states, we set Π*_buckle_* = Π*_ud_* for two models with the same properties and dimensions, where Π*_ud_* represents the potential energy of an un-delaminated state. For fixed values of *μ_f_*/*μ_s_* and *ε_M_*, it is evident that Π*_buckle_* is a monotonically increasing function of Γ/(*μ_s_H_f_*) but Π*_ud_* is a constant (Supplementary Figs S8C, S9C, and S10C). Therefore, the critical value of Γ/(*μ_s_H_f_*) on the phase boundary between the delaminated-buckled state and un-delaminated states is determined by the intersections of the two curves of Π*_buckle_* and Π*_ud_* (Supplementary Fig. S11).

Overall, the three-dimensional phase diagram for growth-induced surface instabilities in film-substrate structures can be understood as follows (Fig. 2). As the mismatch strains in film-substrate structures reach critical values, the initially flat structures can transit into various types of surface instability patterns. If the normalized adhesion energies between the films and substrates are sufficiently high, the preferable patterns are crease and fold for structures with relatively compliant films, but wrinkle, period double and ridge for structures with relatively stiff films. As the normalized adhesion energy decreases to critical values, the un-delaminated patterns transit into the delaminated-buckle patterns.

### Wavelengths and amplitudes of growth-induced surface instabilities

We next study the wavelengths and amplitudes of instability patterns on the phase diagram (Fig. 2). For a film-substrate system with *μ_f_*/*μ_s_* < 1.3, a mismatch strain above 0.35 induces nucleation of scattered creases on the surface of the film; further increasing *ε_M_* leads to a pattern of creases with a wavelength *λ_crease_* (Supplementary Fig. S13). The wavelength of the crease pattern approximately follows a linear relation with *ε_M_* as^48^
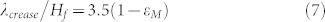
which matches consistently with the current experimental data and the previous study on crease pattern in materials under compression^48^ (Supplementary Fig. S13).

If the modulus ratio of the system *μ_f_*/*μ_s_* is above 1.3, the initially flat surface first bifurcates into the wrinkle phase. The wavelength of the wrinkles at initiation (i.e., just transited from flat state) can be calculated by linear stability analysis and expressed as a function of *μ_f_*/*μ_s_*(see SI and Supplementary Fig. S2D)^49^. For large modulus ratios (i.e., *μ_f_*/*μ_s_* > 10^3^), the wavelength of wrinkles at initiation can be approximated as^50^
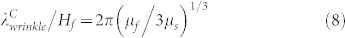


Further increasing the mismatch strain will decrease the wavelengths of the wrinkles. We adopt a modified accordion model to calculate the wavelength of wrinkles with increased mismatch strain. Without loss of generality, let's consider a case that the mismatch strain is induced by the shrinkage of the substrate and the length of the film maintains constant during the process, as illustrate in Supplementary Fig. S14. At a critical mismatch strain, wrinkles with initial wavelength sets in the film-substrate structure. The modified accordion model assumes that the number of undulations in the wrinkles does not change as the substrate further shrinks to increase the mismatch strain above the critical mismatch strain. Therefore, the wavelength of the wrinkles varies according to the mismatch strains at wrinkle initiation and current state, as^37^,^38^,^45^
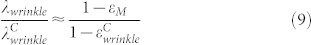
where 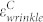 is the critical mismatch strain for wrinkle initiation, given by Eq. 4. The modified accordion model further assumes that the number of undulations in the patterns still maintain the same when the wrinkles transit into folds, period doubles and ridges due to further shrinkage of the substrate. Therefore, we can calculate the wavelengths of folds, period doubles and ridges under various mismatch strains, respectively, as
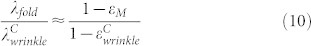

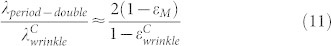

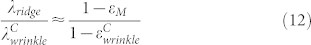
It is noted that the period-double has a wavelength twice of the corresponding wrinkles, as given in Eq. 11.

Once the wavelengths of wrinkles are obtained from Eq. 9, we can further calculate the amplitude of wrinkles by approximating the arc-length of wrinkles at various mismatch strains to be equal to the wavelength of wrinkles at initiation (See Supplementary Fig. S14)^37^,^38^, i.e.,
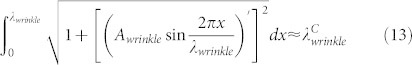
where ()′ is differential operation for *x*.

### Validation of the theoretical models with experiments

We next verify the 3D phase diagram by comparing with experimental results on polymeric film-substrate structures with mismatch strains. We induce the mismatch strain in the film-substrate structure by uniaxially pre-stretching an elastomer substrate, adhering a polymer film on the substrate, and then relaxing the substrate to the original length (see Methods and Fig. 1C). While the shear modulus of the substrate is fixed to be 10.4 *kPa*, the shear modulus of the film is varied from ~3 *kPa* to ~0.8 *GPa*, giving modulus ratio *μ_f_*/*μ_s_* from ~0.3 to ~8 × 10^4^. The adhesion energy between the film and substrate is controlled to vary from 10^−2^ *Jm*^−2^ to 10^3^ *Jm*^−2^ by baking the film-substrate structures at different temperatures (see Methods and Supplementary Fig. S12)^39^. In order to avoid the film-substrate delamination, a very high adhesion energy (*i.e*., Γ > 10^3^ *Jm*^−2^ and Γ/(*μ_s_H_f_*) > 10^3^) is achieved by smearing a thin adhesive layer between the film and substrate^47^,^48^,^51^. Since the adhesive layer is much thinner than the film and its modulus approximates that of the substrate, the adhesive layer does not affect the instability patterns^39^.

We first discuss the five modes of patterns observed in the film-substrate structures with strong adhesion that prevents delamination: (i) If the film is more compliant than the substrate, for example *μ_f_*/*μ_s_* = 0.3 or 0.64, the structure maintains flat under relatively low mismatch strain. When *ε_M_* reaches ~0.36 (for *μ_f_*/*μ_s_* = 0.3 and 0.64), the initially flat surface suddenly forms discrete creases as indicated by arrows in Fig. 3B, which then evolve into periodically distributed creases with the rise of *ε_M_*^12^,^43^. (ii) When the modulus ratio increases to a range of *μ_f_*/*μ_s_* = 1.84 − 7.94 × 10^4^, the initially flat structure first forms wrinkles under moderate mismatch strains (Figs. 3C–3E). As the mismatch strains further increase to critical values, the wrinkles can bifurcate into folds, period doubles or ridges, depending on the modulus ratio. (iii) If the film is slightly stiffer than the substrate with *μ_f_*/*μ_s_* = 1.86, 3.64 or 9.79, the wrinkled surface folds against itself to form creases at some valleys once *ε_M_* reaches ~0.33 (Fig. 3C). With further increase of mismatch strain, all valleys sequentially collapse into folds and further penetrate into the substrate (e.g., *ε_M_* = 0.59 in Fig. 3C). (iv) When the modulus ratio is further increased to a higher range, with *μ_f_*/*μ_s_* = 14.77, 67.24 or 130.74, the wrinkles transit into period-doubles at *ε_M_* ≈ 0.32, by growing the amplitude of one wrinkle at the expense of its neighbors (Fig. 3D). With further increasing *ε_M_*, the crests of period doubles may contact each other to form channels in the valleys (see *ε_M_* = 0.48 in Fig. 3D). (v) If the film is much stiffer than the substrate, for example *μ_f_*/*μ_s_* = 1.59 × 10^3^, 9.11 × 10^3^ or 7.94 × 10^4^, the wrinkles bifurcate into ridge at *ε_M_* ≈ 0.33 (Figs. 3A and 3E). From the comparisons in Fig 3A, it can be seen the observed patterns of creases, wrinkles, folds, period doubles and ridges follow consistently with the prediction of the phase diagram.

**Figure 4:**
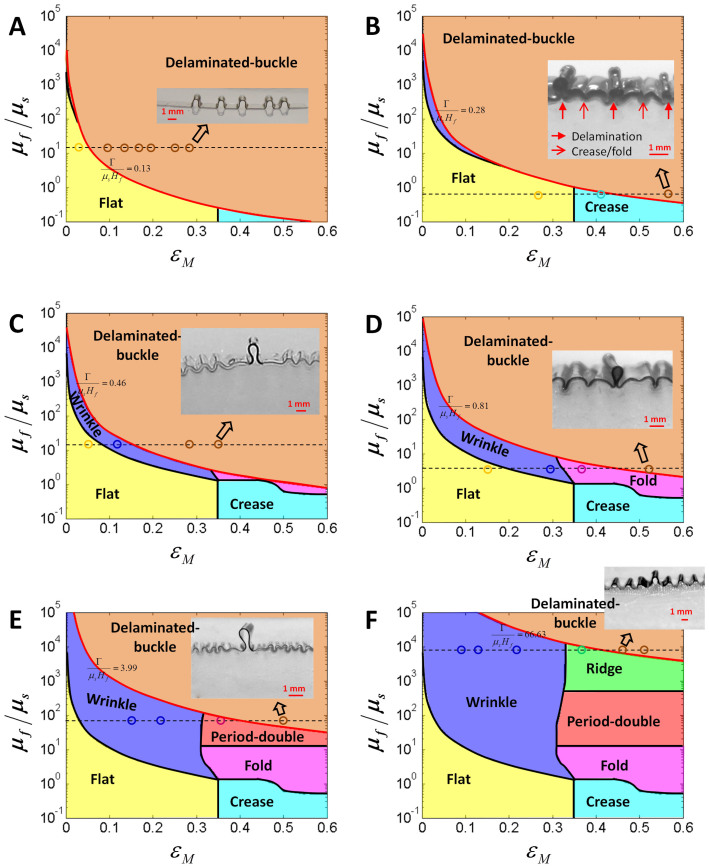
Experimental validation of the phase diagram for instability patterns in film-substrate structures with moderate adhesion energies. Comparison between experimental data and the phase diagrams of surface instability patterns with delamination: (A) flat to delaminated-buckle, (B) crease to delaminated-buckle, (C) wrinkle to delaminated-buckle, (D) fold to delaminated-buckle, (E) period-double to delaminated-buckle, and (F) ridge to delaminated-buckle. The circle markers with different colors in each phase domain represent the observed instability patterns. The inset images in each phase diagram represent the corresponding delaminated-buckle patterns. The two-dimensional phase diagrams are achieved by sectioning the three-dimensional phase diagram at the normalized adhesion energies Γ/(*μ_s_H_f_*) equal to (A) 0.13, (B) 0.28, (C) 0.46, (D) 0.81, (E) 3.99 and (F) 66.63, respectively.

Next, we discuss the observed patterns in film-substrate structures with moderate adhesion energies, which allow delamination between films and substrates. To compare the experimental observations with the calculated phase diagram, we section the three-dimensional phase diagram at various values of normalized adhesion energy, *i.e.*, Γ/(*μ_s_H_f_*) = 0.13, 0.28, 0.46, 0.81, 3.99 and 66.63 (Fig. 4). The phase boundaries between delaminated-buckle and other patterns are highlighted as red curves in these sections. From Fig. 4, it can be seen that the observed transitions of phases with the increase of *ε_M_* indeed follow the calculated phase diagram, for various values of Γ/(*μ_s_H_f_*) and *μ_f_*/*μ_s_*. In particular, the delaminated-buckle can coexist with other instability patterns, and the phase boundaries between delaminated-buckle and other patterns can consistently predict whether delamination occurs in the film-substrate structures.

**Figure 5:**
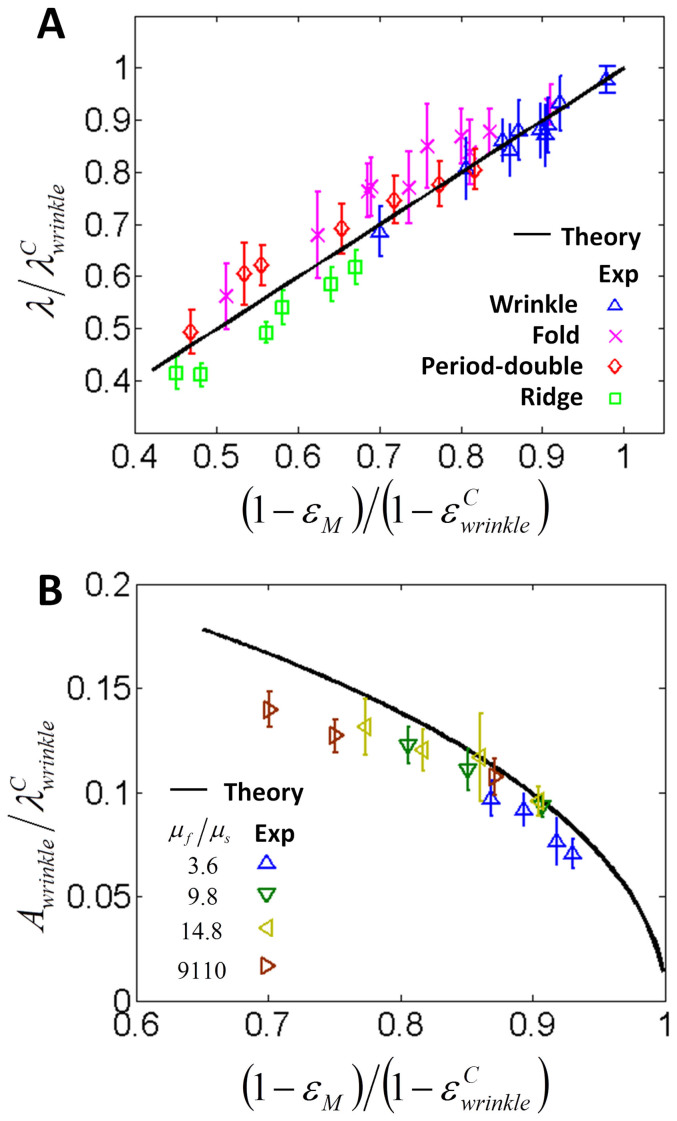
Wavelengths and amplitudes of wrinkles and other instability phases. (A) Wavelengths of wrinkles, folds and ridges, and half wavelength of period-doubles varied with the mismatch strains. (B) Amplitudes of wrinkles varied with the mismatch strains and the modulus ratios.

Finally, the wavelengths and amplitudes of the instability patterns are also validated by our experimental results. As shown in Fig. 5A, the experimentally observed wavelengths of the wrinkles, folds, period-doubles and ridges at varied mismatch strains match consistently with the predictions from Eqs. 9–12. In addition, combining Eq. 13 and 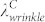 in Eq. 8, we compute the amplitude of the wrinkle for various *μ_f_*/*μ_s_* and *ε_M_* in Fig. 5B. The calculated amplitudes of wrinkles match well with the experimental data for low modulus ratios, and the discrepancies for high modulus ratios are within 10% (Fig. 5B).

## Discussion

**Figure 6:**
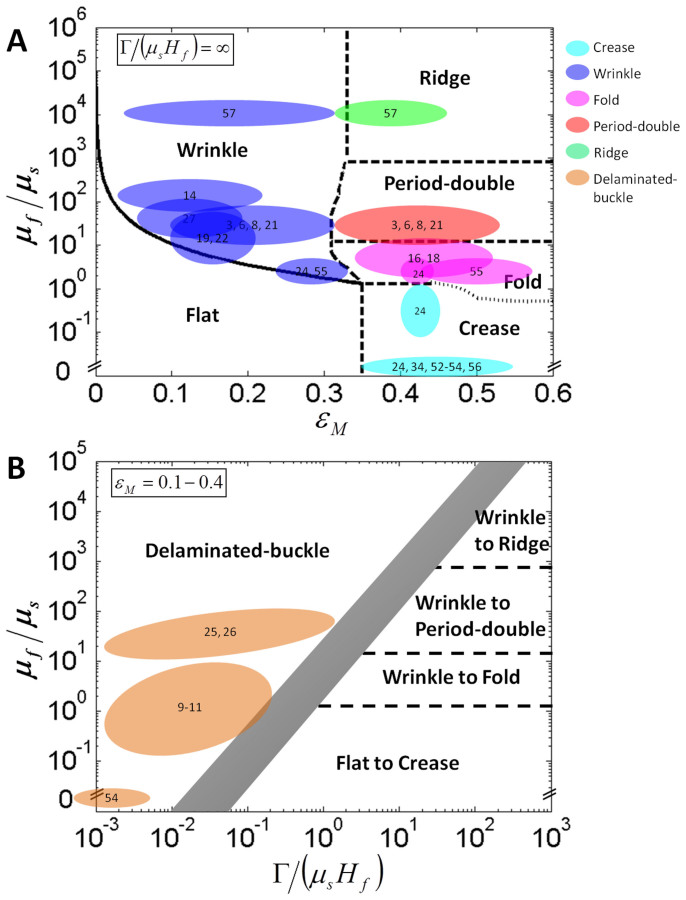
Validation of the phase diagram with reported experimental data. The previous studies on growth/swelling induced surface instabilities for film-substrate structures with (A) high adhesion energies that prevent delamination and (B) moderate adhesion energies that permit delamination. The numbers in the colored domains denote the reference number. The two-dimensional phase diagram in (B) is achieved by stacking the three-dimensional phase diagram in the region of *ε_M_* = 0.1 − 0.4.

The three-dimensional phase diagram is not only validated by our experimental results, but also by reported data on surface instabilities in various biological and biomimetic film-substrate structures. Figure 6 summaries the growth or swelling induced surface instabilities from a number of biological and biomimetic film-substrate systems, including animal tissue growth^14^,^16^,^18^,^21^,^22^, epithelial monolayer growth^19^,^25^,^26^, blood cell growth^27^, fruit growth or shrinkage^3^,^6^,^8^, biofilm growth^9^,^10^,^11^, and swelling of biomimetic hydrogels and elastomers^24^,^34^,^52^,^53^,^54^,^55^,^56^,^57^. The reported or estimated film-substrate modulus ratios, adhesion energies and mismatch strains are summarized in Table 1 (see details in SI).

From Fig. 6, it can be seen the reported surface instability patterns indeed follow the three-dimensional phase diagram. If the film and the substrate are well bonded (Fig. 6A), swelling gels constrained by a rigid substrate on the bottom with *μ_f_*/*μ_s_* < 10^−3^ develop creases on the surfaces^34^,^52^,^53^,^54^,^56^. The growing tissues^14^,^16^,^18^,^19^,^21^,^22^,^25^,^26^, tumors^24^ and blood cells^27^, plant skins^3^,^6^,^8^, and mammalian skins^3^, with *μ_f_*/*μ_s_* in the range of 10^0^–10^4^, generally develop wrinkles in the systems with small mismatch strains, *i.e.*, *ε_M_* = 0.05−0.25; the wrinkles may further bifurcate into folds in the growing tissues^14^,^16^,^18^,^22^ with larger mismatch strains (*ε_M_* > 0.3), or transit into period-double in the mucosal guts^21^ and on severely drying fruits^8^. The transition from wrinkles to folds has also been validated in swelling hydrogel bilayers^24^,^55^. In addition, in a system consisted of a swelling elastomer film on an underlying elastomer substrate (*μ_f_*/*μ_s_* ≈ 10^4^), the transition from wrinkle to ridge has been observed^57^.

If the adhesion energy between the film and substrate is relatively low (Fig. 6B), multiple delamination patterns have been observed in growing biological systems. For example, growing biofilms may delaminate to form buckle regions to facilitate nutrient transportation^9^,^10^,^11^; the epithelial cells may delaminate due to overcrowd or self-metabolism^25^,^26^; the swelling hydrogels loosely bonded on substrates may delaminate to form delaminated-buckles^54^. Since the mismatch strains in these phenomena are generally less than 0.4, we stack the three-dimensional phase diagram in the region of *ε_M_* = 0.1 − 0.4 into a two-dimensional phase diagram (Fig. 6B), where the boundary between delaminated-buckle and other phases is represented by a grey band. From Fig. 6B, it can be seen the reported delaminated-buckle patterns indeed fall in the delaminated-buckle region predicted by the phase diagram.

It should be noted that the phase diagram presented in this paper (Fig. 2) is valid for a wide range of dimensional parameters of growing biological systems. From Fig. 6, Table 1 and Supplementary methods, we can see that the film thickness varies from nanometer to centimeter, the modulus from Pascal to Gigapascal, the adhesion from 10^−2^ *J*/*m*^2^ to 10^3^ *J*/*m*^2^ and the mismatch strain *ε_M_* from 0 to ~0.6. The cases with excessively large film thickness (>m), large modulus ratio (*μ_f_*/*μ_s_* > 10^5^) and large mismatch strain (>0.6) have not been considered in the phase diagram.

In summary, we present a three-dimensional phase diagram that can quantitatively predict various modes of growth-induced surface instabilities in biological film-substrate structures. By combining theory, computation and experiment, we show that the initially flat biological layers can systematically transform into instability patterns of wrinkle, crease, fold, period-double, ridge, delaminated-buckle and their coexistences, depending on three non-dimensional parameters: mismatch compressive strain, film-substrate modulus ratio, and normalized adhesion energy. The three-dimensional phase diagram offers a unified model for understanding morphogenesis in biological film-substrate structures on a mechanical base. The method for constructing the phase diagram opens new venues to study the formation of more complicated patterns, for example, in multi-layer structures, structures with intrinsic surface curvatures^6^,^58^, and inhomogeneous and/or anisotropic structures^6^,^8^,^16^,^18^,^59^,^60^. To the end, the phase diagram can potentially guide the rational design of a variety of biomimetic topographical-structures for engineering applications as diverse as disease therapy^22^,^24^, active cell culture^34^, biofouling management^35^, tunable superhydrophobicity^36^ and flexible electronics^37^,^38^,^61^.

## Methods

### Materials

We use an elastomer film (Ecoflex 0010, Smooth-on, USA) with the thickness ~5 mm and the modulus ~10.4 *kPa* as the substrate. The shear modulus of Ecoflex is measured by fitting stress *vs*. strain data from uniaxial tensile tests to the incompressible neo-Hookean law (Micro-Strain Analyzer, TA Instruments, USA). We choose films with a large range of moduli, from ~3 *kPa* to ~0.8 *Gpa*. Below 2 *Mpa*, a silicone elastomer Sylgard 184 (Dow Corning, USA) is spin-casted into thin films with thickness ~200 *μm*. The shear modulus of Sylgard is varied from 3.1 *kPa* to 1.4 *MPa* by changing its cross-linker concentration. Above 2 *Mpa*, natural rubber (~16 *Mpa*, 50 *μm*, McMaster-Carr, USA), low density polyethylene (LPE, ~96 *Mpa*, 25 *μm*, McMaster-Carr, USA) and Kapton (0.83 *Gpa*, 25 *μm*, McMaster-Carr, USA) are used to act as the films.

### Experimental procedure

To simply induce a mismatch strain between the film and substrate, we first uniaxially pre-stretched the substrate to a prescribed ratio of *L_f_*/*L_s_* (Fig. 1Cii). The Sylgard film is then carefully attached on the pre-stretched substrate by uniformly pressing it on the substrate with two rigid plates, and the bilayer then is baked at an oven with a controlled temperature for 10 min. Thereafter, the pre-stretched substrate is gradually relaxed to the original length with a strain rate around 1 × 10^−3^ *s*^−1^. The surface morphology is captured by a camera (Cannon, USA) with a tilted angel 20°. To enhance the film-substrate adhesion, a very thin layer of uncured Ecoflex can also be smeared on the substrate prior to attaching the films^48^,^51^.

### Measurement of the adhesion energy

The adhesion energy between the film and substrate is measured with the peeling test (Supplementary Fig. S12)^62^. A strip of Sylgard film with width *b* is carefully attached on a prestretched substrate, and the bilayer then is baked at an oven with a controlled temperature for 10 min. A force *F* is then applied to the Sylgard film to peel off the substrate along an angle *θ* at a low peeling rate 1 × 10^−4^ *m*·*s*^−1^. The adhesion energy is calculated by Γ = (1 − cos *θ* + *ε_d_*/2)*F*/*b*, where *ε_d_* is the strain in the detached section of the Sylgard film.

### Finite element calculations

The finite element calculations (Supplementary Figs S3–S11) are implemented by software ABAQUS 10.1. The film and the substrate are taken to be incompressible neo-Hookean materials with shear moduli *μ_f_* and *μ_s_* respectively. The thickness of the substrate is much larger than that of the film (>20 times). Three methods are used to induce mismatch compressive strains in the films: In the first method, a film-substrate laminate is prestretched by a ratio of *L_f_*/*L_s_*, and then the strain in the film is released, followed by subsequently relaxing the laminate to the original length *L_s_* (Supplementary Figs S3, S4, S5A, S6, S7A and S8–S11). The second method involves swelling of the film in the horizontal direction (Supplementary Figs S5B and S7B), while the third method is shrinking the substrate in the horizontal direction (Supplementary Figs S5C and S7C). Small force perturbations are introduced to facilitate the initiation of instabilities. The calculation models are discretized by CPE6MH elements, and the result accuracy is ascertained through mesh refinement studies.

## Author Contributions

Q.W. and X.Z. designed the research. Q.W. performed the experiments. Q.W. and X.Z. developed and analyzed the theoretical models and numerical calculations, interpreted the results, and wrote the manuscript.

## Supplementary Material

Supplementary Information Supplementary information

## Figures and Tables

**Table 1:** Reported and estimated parameters of growth/swelling film-substrate systems with various surface instabilities in published studies

Reference	System	Instability type	*ε_M_*	μ*_f_*/μ*_s_*	Γ/(*μ_s_H_f_*)</emph>
16,18	tissue	fold	0.29 − 0.4	1.5 − 15	>10^3^
14	tissue	wrinkle	0.05 − 0.18	100 − 300	>10^3^
22	tissue	wrinkle	0.1 − 0.15	5 − 50	>10^3^
21	tissue	wrinkle, period-double	0.05 − 0.45	10 − 100	>10^3^
19	epithelia	wrinkle	0.05 − 0.2	~25	>10^3^
25,26	epithelia	delaminated-buckle	0.1 − 0.22	1 − 100	10^−3^ − 1
27	blood cell	wrinkle	0.05 − 0.2	50 − 100	>10^3^
9,10,11	biofilm	delaminated-buckle	0 − 0.4	0.1 − 10	10^−3^ − 0.2
3,6,8	fruit	wrinkle, period-double	0.1 − 0.4	10 − 100	>10^3^
34,52,53,56	hydrogel	crease	0.32 − 0.5	<10^−3^	>10^3^
24	hydrogel	crease, wrinkle, fold	0.25 − 0.45	1 − 20	>10^3^
55	hydrogel	wrinkle, fold	0.3 − 0.55	~25	>10^3^
54	Sylgard	crease, delaminated-buckle	0.3 − 0.51	<10^−3^	5 × 10^−5^ − 5 × 10^−3^
57	Sylgard	wrinkle, ridge	0.05 − 0.45	~10^4^	>10^3^
