# Supplementary material for: A three-dimensional phase diagram of growth-induced surface instabilities
Source: Sci Rep. 2015 Mar 9;5:8887. doi: 10.1038/srep08887 (PMC4352857; doi:10.1038/srep08887)
Supplement: Supplementary Information [file srep08887-s1.pdf]

Supplementary Information for

A Three-Dimensional Phase Diagram of Growth-Induced Surface Instabilities

Qiming Wang[1,2], Xuanhe Zhao[1,2,3*]

[1]*Soft Active Materials Laboratory, Department of Mechanical Engineering, Massachusetts Institute of Technology, Cambridge, Massachusetts 02139, USA.* [2]*Department of Mechanical Engineering and Materials Science, Duke University, Durham, North Carolina 27708, USA.* [3]*Department of Civil and Environmental Engineering, Massachusetts Institute of Technology, Cambridge, MA 02139, USA.*

*Email: zhaox@mit.edu

Supplementary figures

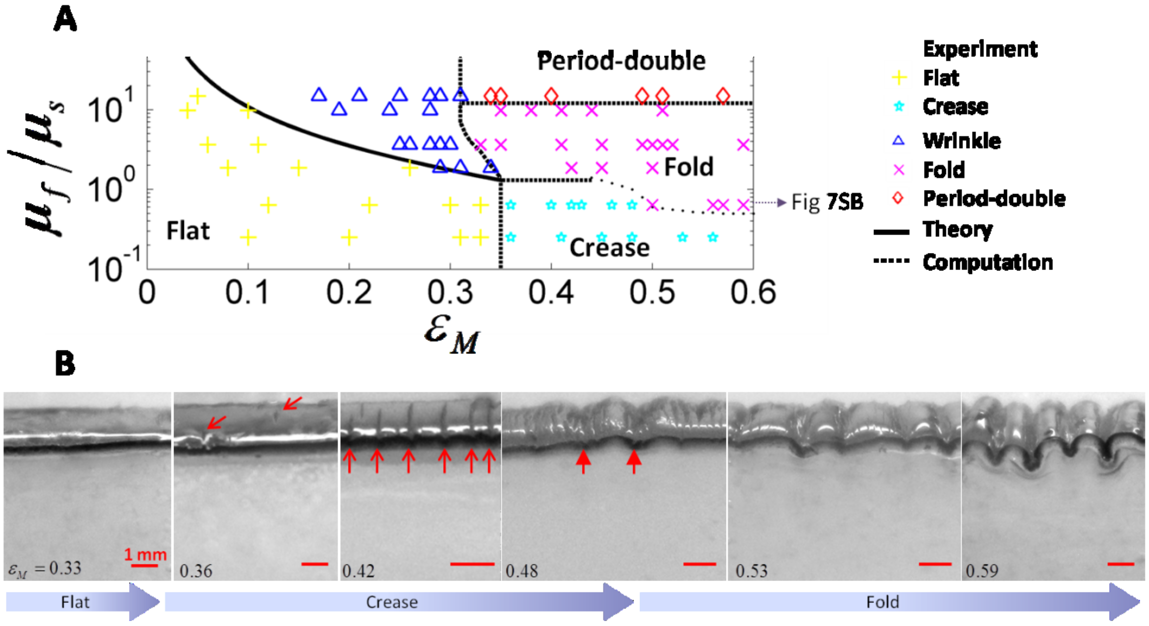

Supplementary Figure S1 | The transition process from creases to folds. (A) Comparison between the calculated phase diagram and experimental results for the transition from creases to folds. (B) Sequences of experimental images to show the transition process from creases to folds with increasing mismatch strain.

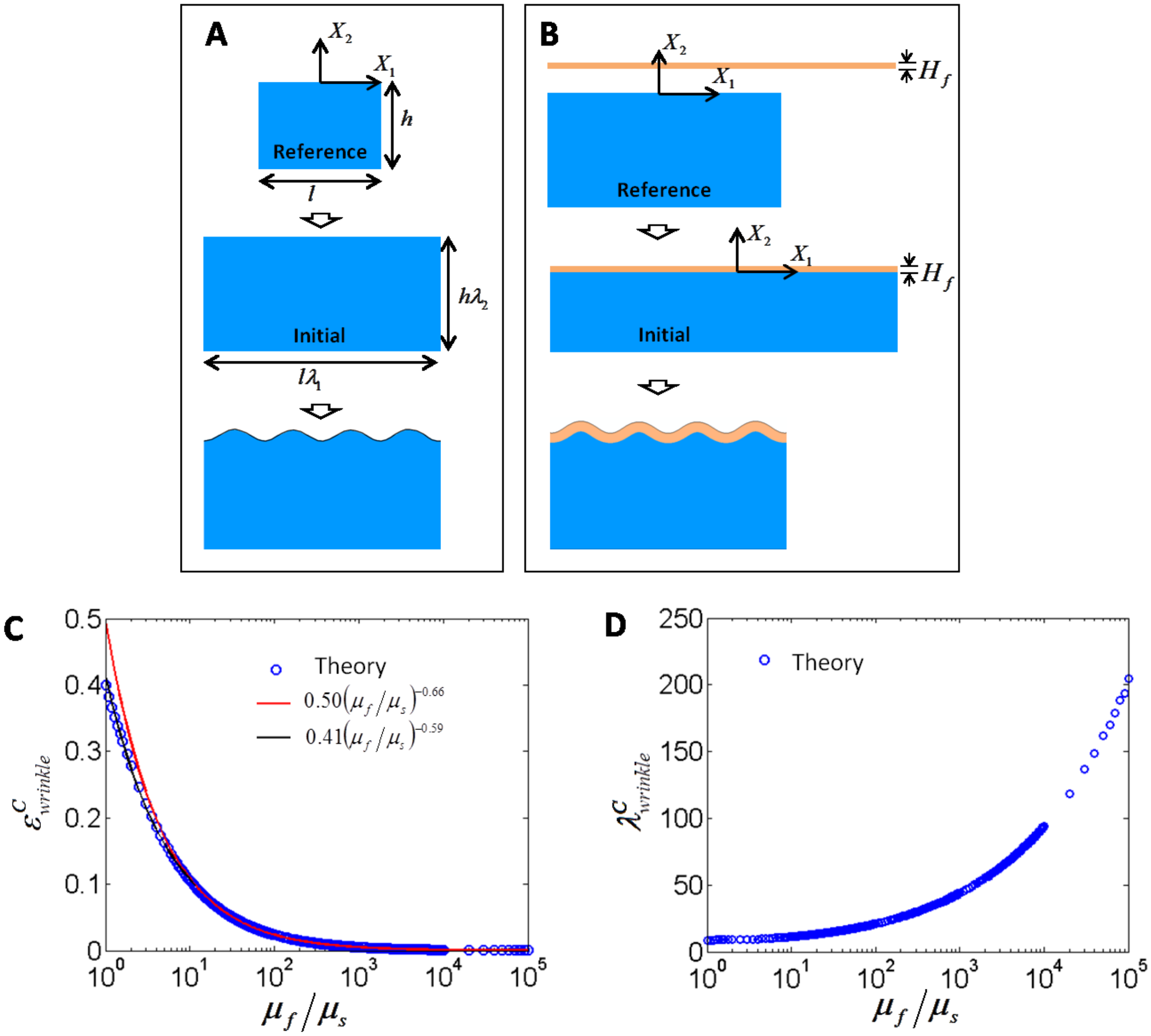

Supplementary Figure S2 | Calculation of the phase boundary between the flat and wrinkled states. Schematics of (A) a neo-Hookean block and (B) a neo-Hookean film-substrate structure. (C) Calculated critical mismatch strain and (D) critical wavelength between flat and wrinkled states as a function of modulus ratio μ_f/μ_s .

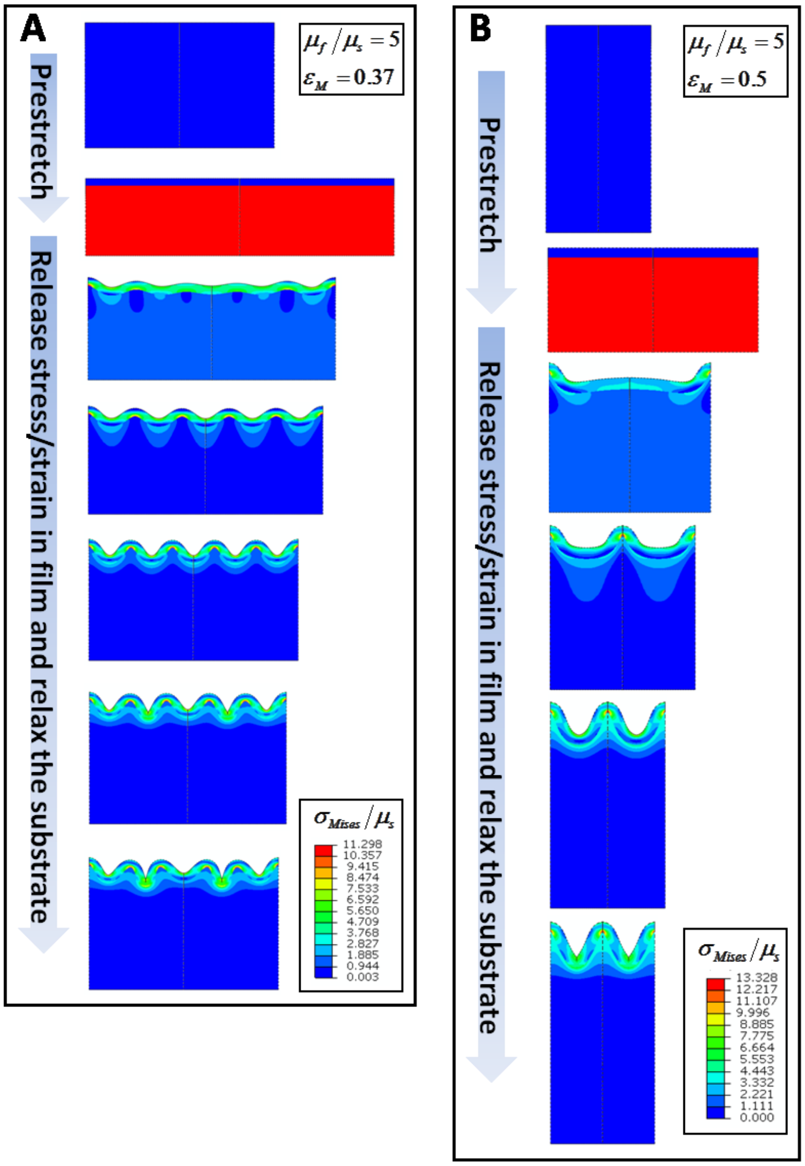

Supplementary Figure S3 | Examples of finite-element models that give the formation of folds in film-substrate structures: (A) $\varepsilon_M = 0.37$, $\mu_f/\mu_s = 5$ **and (B)** $\varepsilon_M = 0.5$, $\mu_f/\mu_s = 5$.

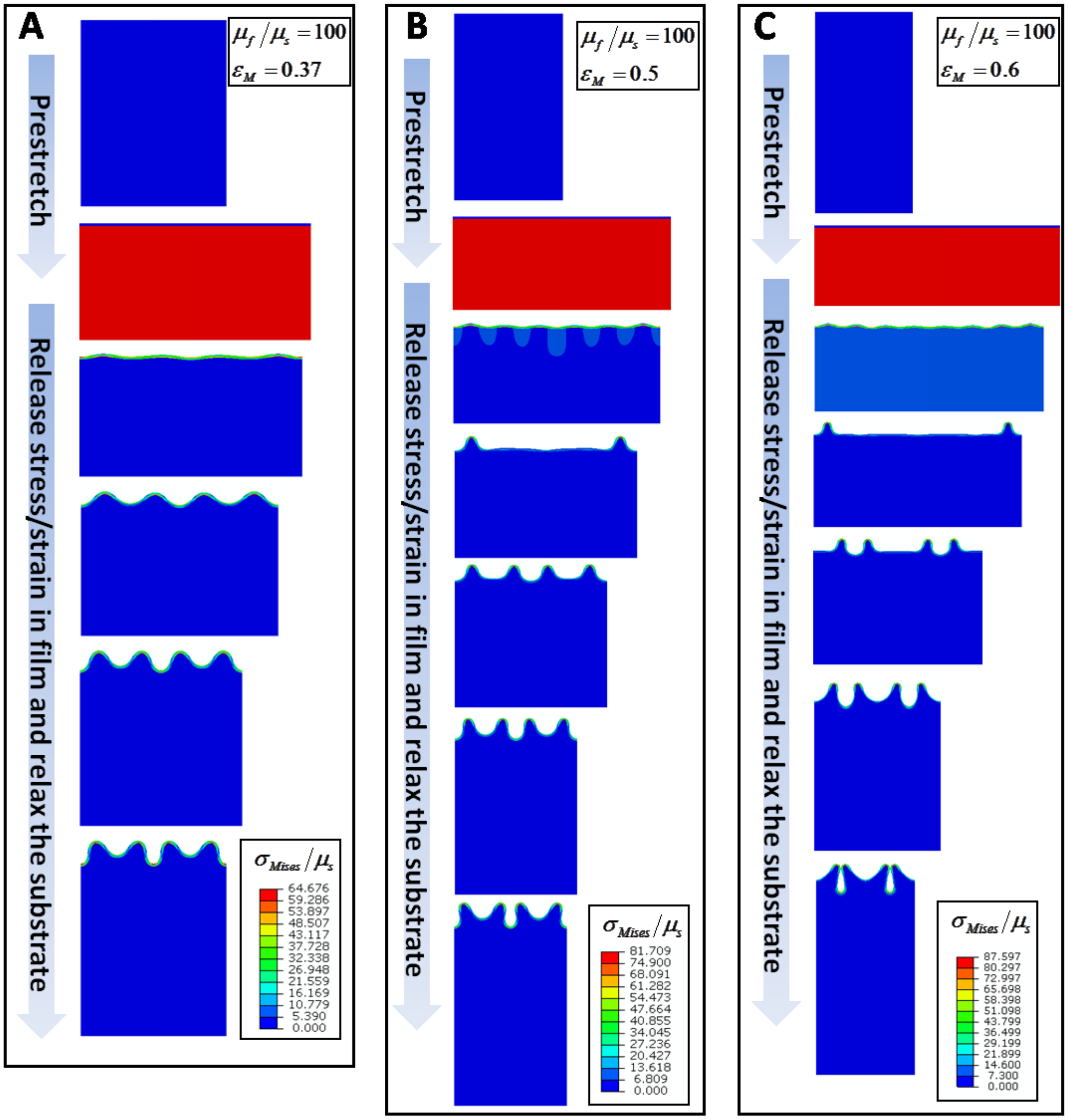

Supplementary Figure S4 | Examples of finite-element models that give the formation of period-doubles in film-substrate structures: (A) $\varepsilon_M = 0.37$, $\mu_f/\mu_s = 100$; **(B)** $\varepsilon_M = 0.5$, $\mu_f/\mu_s = 100$; **and (C)** $\varepsilon_M = 0.6$, $\mu_f/\mu_s = 100$.

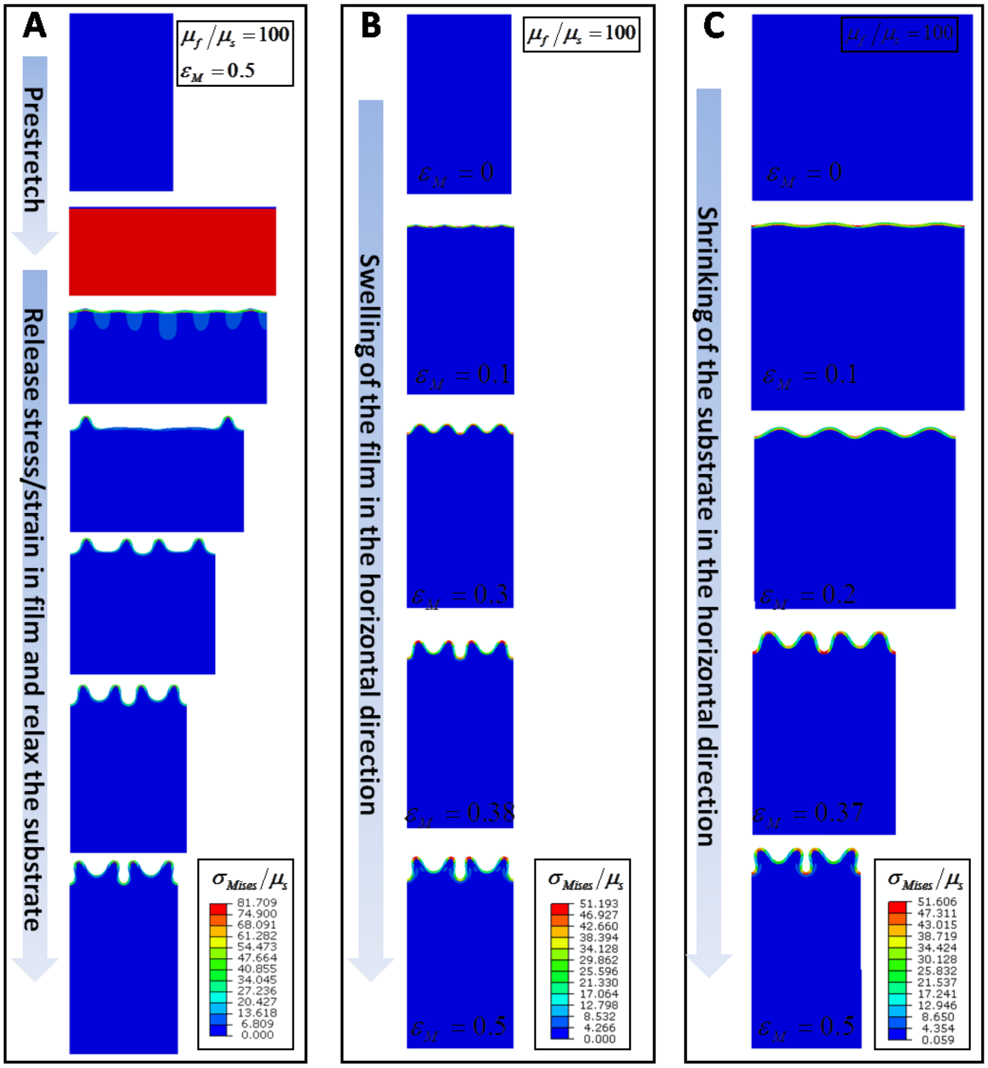

Supplementary Figure S5 | Examples of finite-element models that give the formation of period-doubles in film-substrate structures by following different paths to induce the mismatch strains: (A) substrate pre-stretching and relaxing, (B) film swelling in the horizontal direction, and (C) substrate shrinking in the horizontal direction.

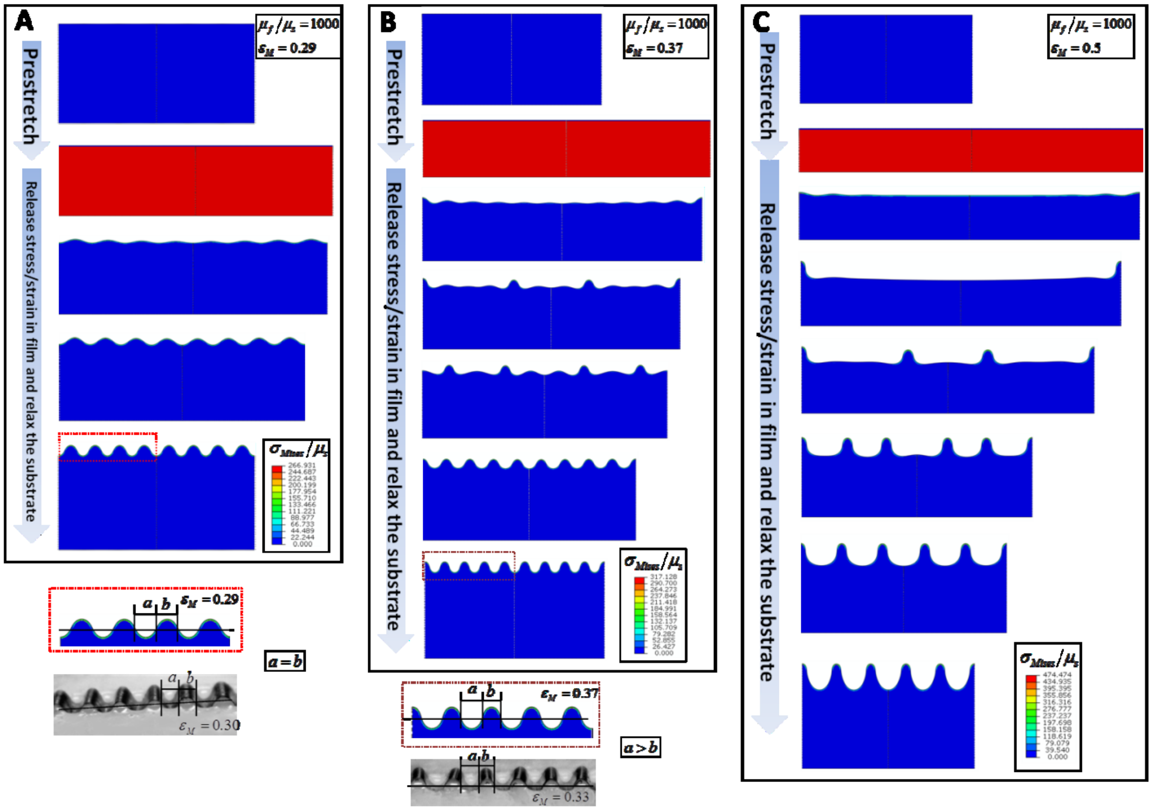

Supplementary Figure S6 | Examples of finite-element models that give the formation of wrinkles in film-substrate structure with (A) $\varepsilon_M = 0.29$, $\mu_f/\mu_s = 1000$; and ridges with (B) $\varepsilon_M = 0.37$, $\mu_f/\mu_s = 1000$and (C) $\varepsilon_M = 0.5$, $\mu_f/\mu_s = 1000$. The half-height-lengths of adjacent wrinkles are equal (a=b) as shown in (A); while the half-height-lengths of adjacent ridges are unequal (b<a) as shown in (B).

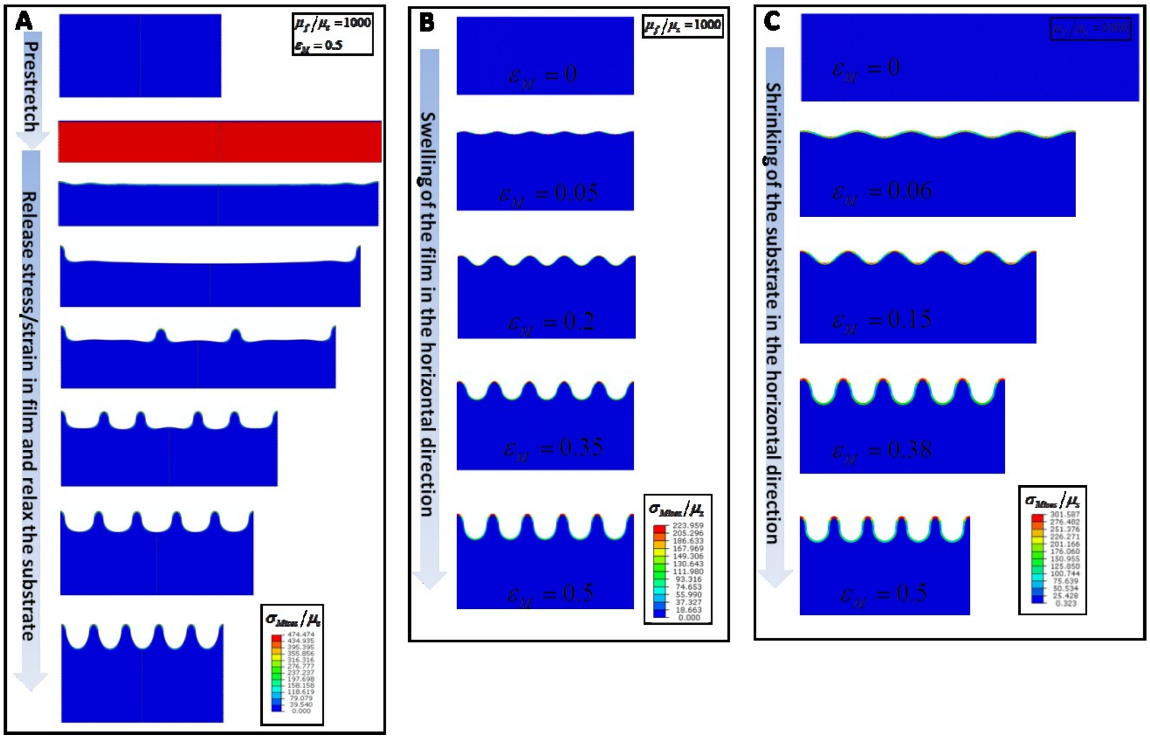

Supplementary Figure S7 | Examples of finite-element models that give the formation of ridges in film-substrate structures by following different paths to induce the mismatch strains: (A) substrate pre-stretching and relaxing, (B) film swelling in the horizontal direction, and (C) substrate shrinking in the horizontal direction.

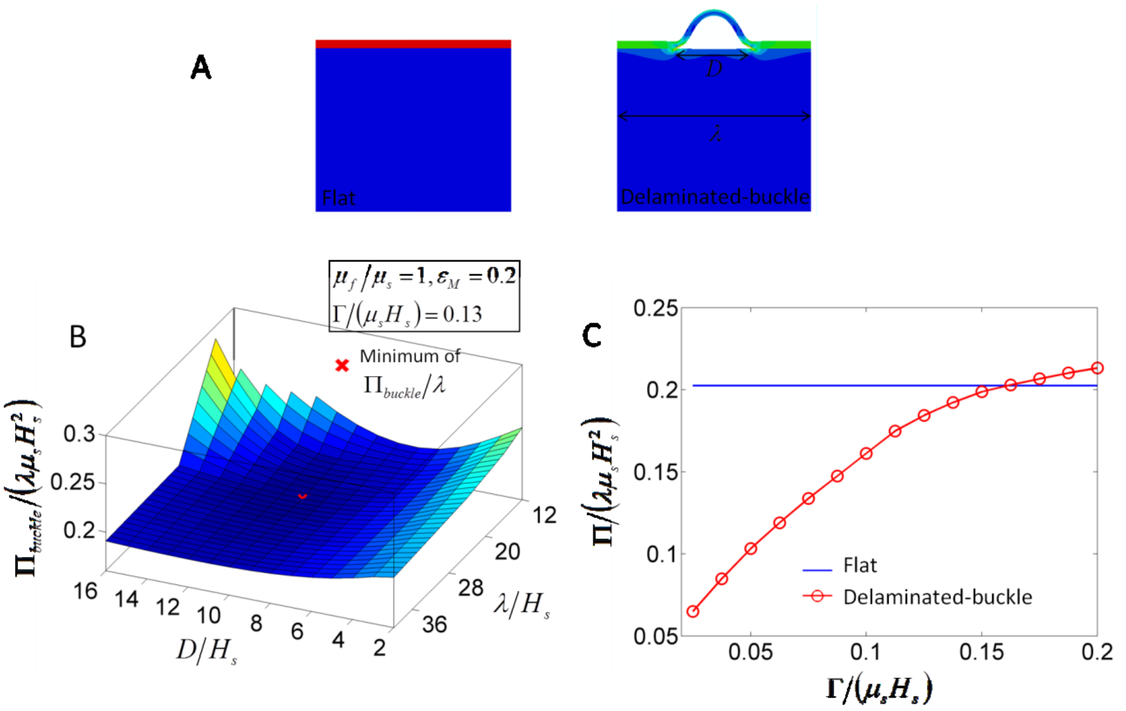

Supplementary Figure S8 | Calculation of the phase boundary between the flat and delaminated-buckled states. (A) Finite-element model of a film-substrate structure at flat and delaminated-buckled states with $\mu_f/\mu_s=1$ and $\varepsilon_M=0.2$. (B) The potential energy per wavelength of the delaminated-buckled state as a function of delamination length D and wavelength λ. The minimum of potential energy per wavelength of the structure is determined by $\partial(\Pi_{buckle}/\lambda)/\partial\lambda=0$ and $\partial(\Pi_{buckle}/\lambda)/\partial D=0$. (C) The potential energy per wavelength of the structure at flat and delaminated-buckled states as functions of the film-substrate adhesion energy. The intersection point ($\Gamma/(\mu_s H_f)=0.158$) between two lines denotes the phase boundary between the flat state and delaminated-buckled state. Therefore, when $\Gamma/(\mu_s H_f)<0.158$, the delaminated-buckled state is the current state of the structure with $\mu_f/\mu_s=1$ and $\varepsilon_M=0.2$; when $\Gamma/(\mu_s H_f)>0.158$, the flat state is the current state.

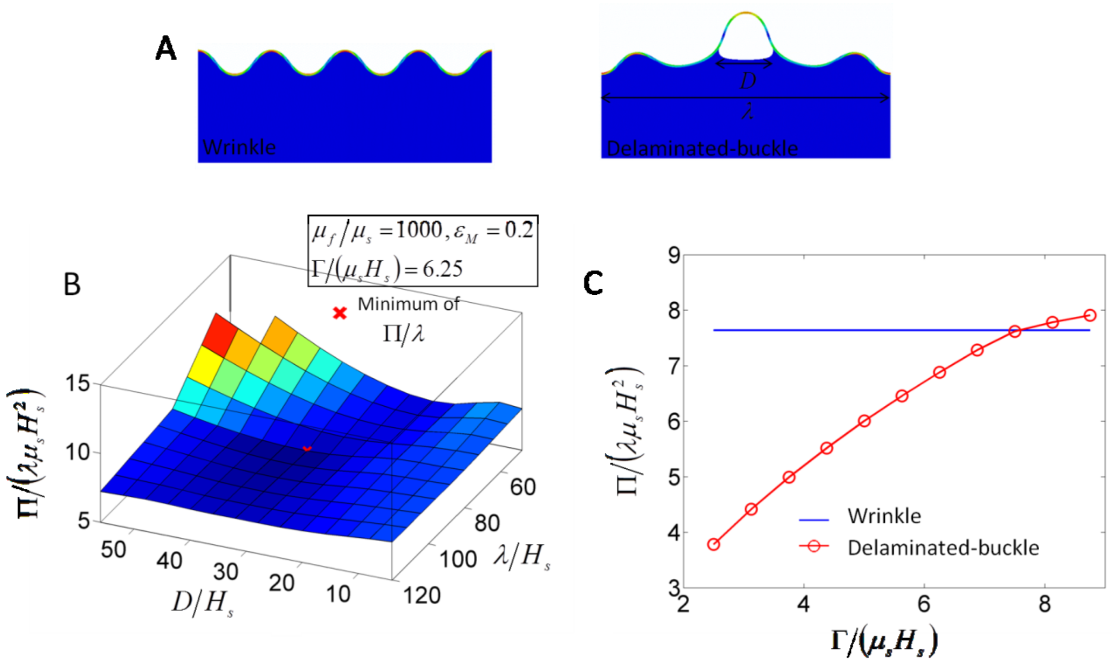

Supplementary Figure S9 | Calculation of the phase boundary between the wrinkled and delaminated-buckled states. (A) Finite-element model of a film-substrate structure at wrinkled and delaminated-buckled states with $\mu_f/\mu_s = 1000$and $\varepsilon_M = 0.2$. (B) The potential energy per wavelength of the delaminated-buckled state as a function of delamination length D and wavelength λ. The minimum of potential energy per wavelength of the structure is determined by $\partial(\Pi_{buckle}/\lambda)/\partial\lambda = 0$and $\partial(\Pi_{buckle}/\lambda)/\partial D = 0$. (C) The potential energy per wavelength of the structure at wrinkled and delaminated-buckled states as functions of the film-substrate adhesion energy. The intersection point ($\Gamma/(\mu_s H_f) = 7.5$) between two lines denotes the phase boundary between the flat state and delaminated-buckled state. Therefore, when $\Gamma/(\mu_s H_f) < 7.5$, the delaminated-buckled state is the current state of the structure with $\mu_f/\mu_s = 1000$and $\varepsilon_M = 0.2$; when $\Gamma/(\mu_s H_f) > 7.5$, the wrinkled state is the current state.

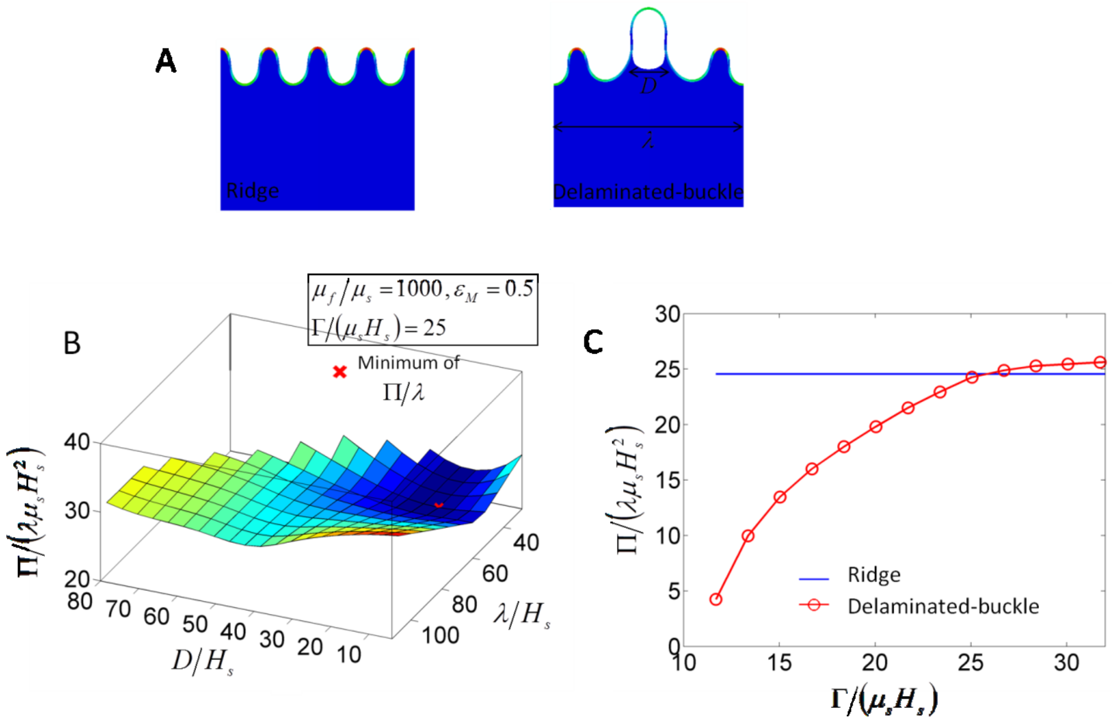

Supplementary Figure S10 | Calculation of the phase boundary between the ridged and delaminated-buckled states. (A) Finite-element model of a film-substrate structure at ridged state and delaminated-buckled state with $\mu_f/\mu_s = 1000$ and $\varepsilon_M = 0.5$. (B) The potential energy per wavelength of the delaminated-buckled state as a function of delamination length D and wavelength λ. The minimum of potential energy per wavelength of the structure is determined by $\partial(\Pi_{buckle}/\lambda)/\partial\lambda = 0$ and $\partial(\Pi_{buckle}/\lambda)/\partial D = 0$. (C) The potential energy per wavelength of the structure at ridged and delaminated-buckled states as functions of the film-substrate adhesion energy. The intersection point ($\Gamma/(\mu_s H_f) = 25$) between two lines denotes the phase boundary between the ridged state and delaminated-buckled state. Therefore, when $\Gamma/(\mu_s H_f) < 25$, delaminated-buckled state is the current state of the structure with $\mu_f/\mu_s = 1000$ and $\varepsilon_M = 0.5$; when $\Gamma/(\mu_s H_f) > 25$, the ridged state is the current state.

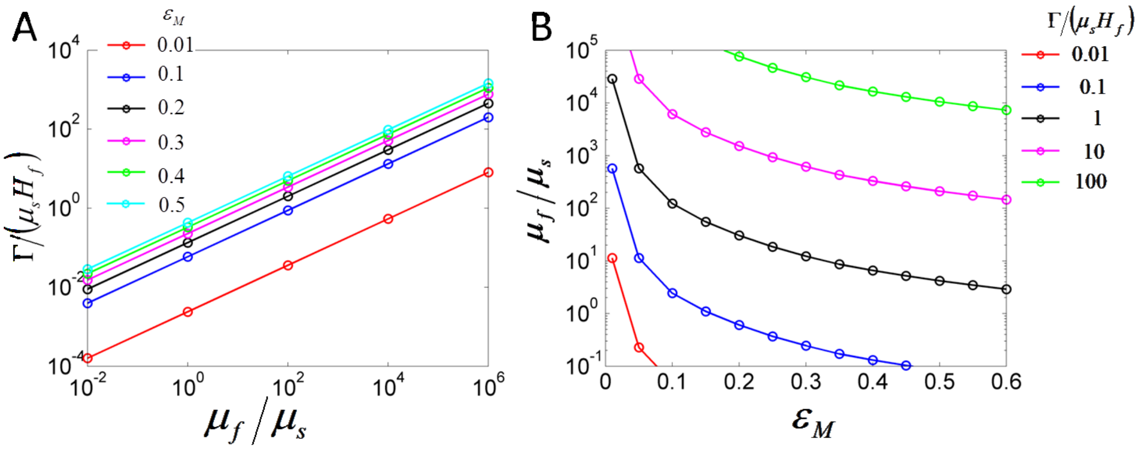

Supplementary Figure S11 | Calculation of the phase boundary between the delaminated-buckled state and un-delaminated state. (A) The calculated critical values of normalized adhesion energy $\Gamma/(\mu_s H_f)$ on the phase boundary between the delaminated-buckled state and un-delaminated state as functions of modulus ratio and mismatch strain. (B) Re-plot of (A) as the calculated critical values of modulus ratio on the phase boundary as functions of mismatch strain and normalized adhesion energy.

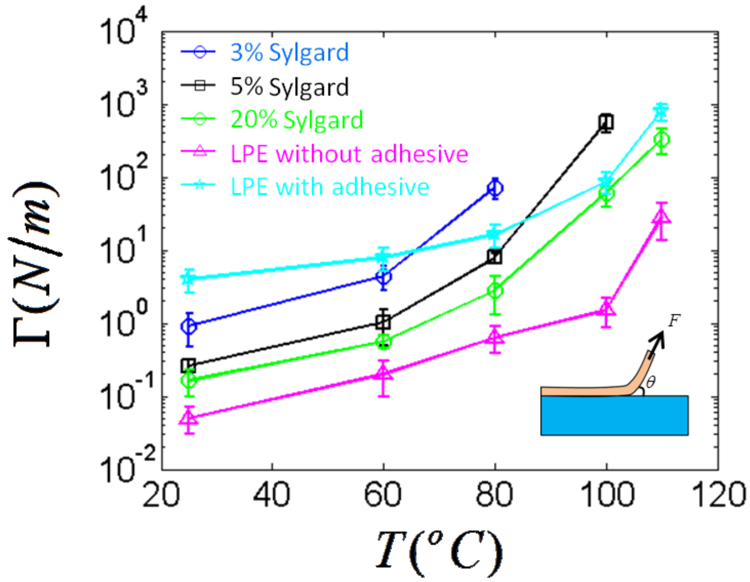

Supplementary Figure S12 | The measured adhesion energy between different films and Ecoflex substrates for different baking temperatures. The adhesive layer is a very thin layer of uncured Ecoflex smeared on the substrate prior to attaching the film.

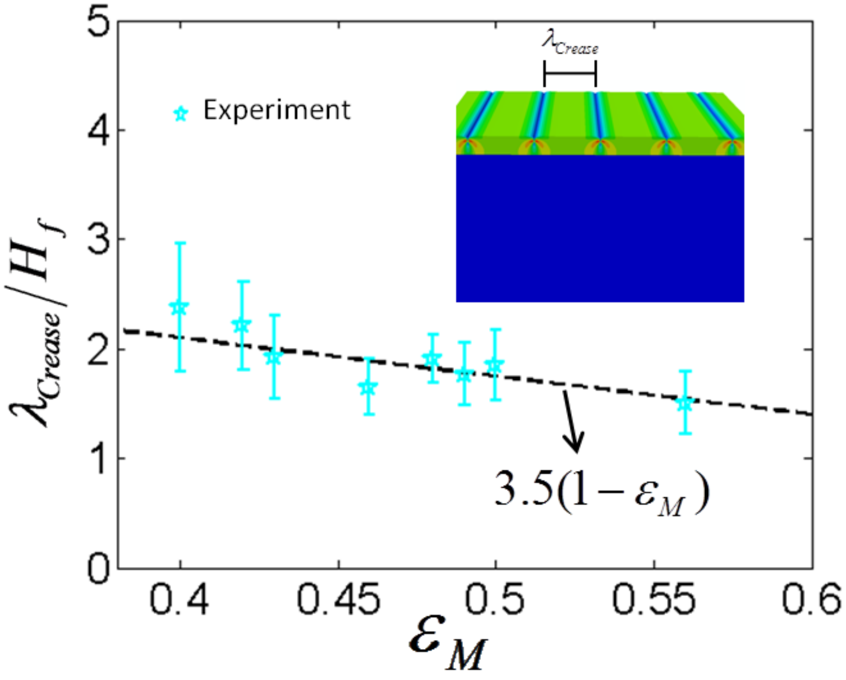

Supplementary Figure S13| Wavelength of creases varied with mismatch strains.

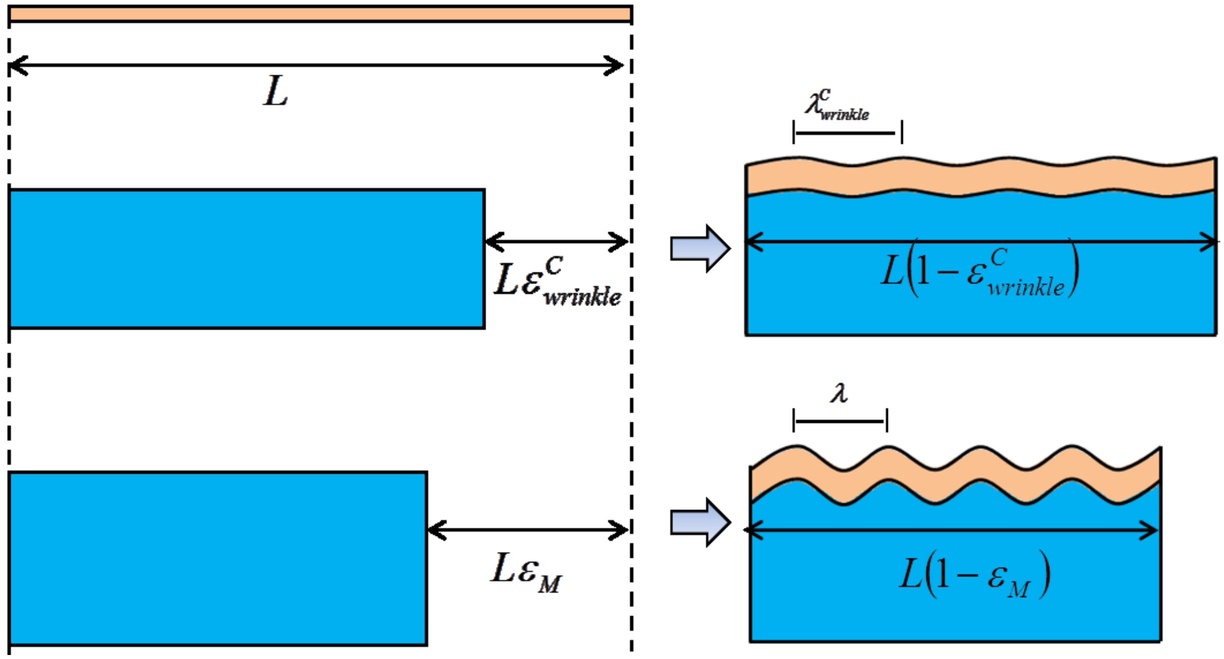

Supplementary Figure S14| Schematics of the modified accordion model. As the mismatch strain increases due to the shrinkage of substrate, the number of undulations in wrinkles maintains constant but the wavelength of the wrinkles decreases accordingly.

Supplementary Methods

Analysis of wrinkling instability

Following Hutchinson and Cao [1-3] and Koiter [4], a stability analysis for the mismatch-strain-induced wrinkling instability is re-derived here to calculate the critical mismatch strain for flat-wrinkling transition in film-substrate systems with varied modulus ratio μ_f / μ_s .

Base solutions of a single neo-Hookean block

We first consider a rectangular incompressible neo-Hookean block, and take its undeformed state as the reference state in the Lagrangian coordinate X_1, X_2 and X_3 as shown in **Supplementary Figure S2A**. The top surface of the block is at $X_2=0$. The block is then homogeneously deformed by stretches λ_1 , λ_2 and λ_3 along three axes, where λ_3 will be fixed in future plane-strain deformation. This homogeneously deformed state is denoted as the initial state of the block.

Next, we will perturb this initial state with small and periodic deformation. Assuming the solutions we seek follow periodic forms with a wavelength l in the reference state in X_1 direction, we take the analysis domain in X_1 direction as $0 \le X_1 \le l$. Since the thickness of the block in X_2 direction is h at the reference state, the analysis domain in X_2 direction is $-h \le X_2 \le 0$.

The deformation gradient tensor for the block at the initial state is

$$\mathbf{F}^0 = \begin{bmatrix} \lambda_1 & & \\ & \lambda_2 & \\ & & \lambda_3 \end{bmatrix} \tag{S1}$$

The strain energy density of this initial state is

$$\varphi_0 = \frac{1}{2}\mu\left(tr\mathbf{F}^{\mathbf{0T}}\mathbf{F}^{\mathbf{0}} - 3\right) = \frac{1}{2}\mu\left(\lambda_1^2 + \lambda_2^2 + \lambda_3^2 - 3\right) \tag{S2}$$

where μ is the shear modulus of the neo-Hookean block. When an incremental displacement U_1 and U_2 is imposed along X_1 and X_2 directions onto the initial state, the resultant deformation gradient of the current state can be expressed as

$$\mathbf{F} = \begin{bmatrix} \lambda_1 + U_{1,1} & U_{1,2} & 0 \\ U_{2,1} & \lambda_2 + U_{2,2} & 0 \\ 0 & 0 & \lambda_3 \end{bmatrix} \tag{S3}$$

where $U_{1,1} = \partial U_1 / \partial X_1$, $U_{2,2} = \partial U_2 / \partial X_2$, $U_{1,2} = \partial U_1 / \partial X_2$ and $U_{2,1} = \partial U_2 / \partial X_1$. The strain energy density of the current state is

$$\varphi = \frac{1}{2}\mu\left(tr\mathbf{F}^{\mathbf{T}}\mathbf{F} - 3\right) = \frac{1}{2}\mu\left[\left(\lambda_1 + U_{1,1}\right)^2 + \left(\lambda_2 + U_{2,2}\right)^2 + \left(U_{2,1}\right)^2 + \left(U_{1,2}\right)^2 + \lambda_3^2 - 3\right] \tag{S4}$$

The difference in strain energy densities between the current and initial states is

$$\varphi - \varphi_0 = \mu\left[\lambda_1 U_{1,1} + \lambda_2 U_{2,2} + \frac{1}{2}\left(U_{1,1}^2 + U_{2,2}^2 + U_{2,1}^2 + U_{1,2}^2\right)\right] \tag{S5}$$

The incompressibility condition, $\det(\mathbf{F}) = 1$, further imposes

$$\Omega(\lambda, U) = \lambda_2 U_{1,1} + \lambda_1 U_{2,2} + U_{1,1}U_{2,2} - U_{2,1}U_{1,2} = 0 \tag{S6}$$

Therefore, the potential energy difference between current and initial states can be expressed as

$$\Phi = \mu\int_0^l\int_{-h}^0 \begin{bmatrix} \lambda_1 U_{1,1} + \lambda_2 U_{2,2} + \frac{1}{2}\left(U_{1,1}^2 + U_{2,2}^2 + U_{2,1}^2 + U_{1,2}^2\right) \\ - q\left(\lambda_2 U_{1,1} + \lambda_1 U_{2,2} + U_{1,1}U_{2,2} - U_{2,1}U_{1,2}\right) \end{bmatrix} dX_1 dX_2 \tag{S7}$$

where $q(X_1, X_2)$ is a Lagrangian multiplier.

To eliminate terms linear in U_i (i=1, 2) in **Supplementary Eq. S7**, we denote $q = r + Q$ with $r \equiv \lambda_2/\lambda_1$, where $Q(X_1, X_2)$ has the same periodicity as U_i. We further assume U_i follows a periodic form with a wavelength l in X_1 direction, so $U_{1,1}$ integrates to zero by periodicity. Consequently, we obtain

$$\Phi = \mu \int_0^l \int_{-h}^0 I(\lambda_i, U_i, Q, r) dX_1 dX_2 \tag{S8}$$

where

$$I = \frac{1}{2}\left(U_{1,1}^2 + U_{2,2}^2 + U_{2,1}^2 + U_{1,2}^2\right) - Q\left(\lambda_2 U_{1,1} + \lambda_1 U_{2,2}\right) - (r + Q)\left(U_{1,1}U_{2,2} - U_{2,1}U_{1,2}\right) \tag{S9}$$

By using variation method, we vary U_1, U_2 and Q by η, ζ and δQ and obtain

$$\begin{aligned} \delta I = &\left(U_{1,1}\eta_{,1} + U_{2,2}\zeta_{,2} + U_{2,1}\zeta_{,1} + U_{1,2}\eta_{,2}\right) - \delta Q\left(\lambda_2 U_{1,1} + \lambda_1 U_{2,2} + U_{1,1}U_{2,2} - U_{2,1}U_{1,2}\right) \\ &- Q\left(\lambda_2 \eta_{,1} + \lambda_1 \zeta_{,2}\right) - (r + Q)\left(U_{1,1}\zeta_{,2} + \eta_{,1}U_{2,2} - \zeta_{,1}U_{1,2} - U_{2,1}\eta_{,2}\right) \end{aligned} \tag{S10}$$

We express the variation of the potential energy difference as

$$\delta\Phi = \mu \int_0^l \int_{-h}^0 \delta I dX_1 dX_2 = 0 \tag{S11}$$

Through integration by parts, we reach

$$\begin{aligned} \int_0^l \int_{-h}^0 \delta I dX_1 dX_2 = &-\int_0^l \int_{-h}^0 \eta\left[U_{1,11} + U_{1,22} - \left(Q_{,1}U_{2,2} - Q_{,2}U_{2,1}\right) - \lambda_2 Q_{,1}\right] dX_1 dX_2 - \\ &\int_0^l \int_{-h}^0 \zeta\left[U_{2,11} + U_{2,22} - \left(Q_{,1}U_{1,2} - Q_{,2}U_{1,1}\right) - \lambda_1 Q_{,2}\right] dX_1 dX_2 - \\ &\int_0^l \int_{-h}^0 \delta Q\left[\lambda_2 U_{1,1} + \lambda_1 U_{2,2} + U_{1,1}U_{2,2} - U_{1,2}U_{2,1}\right] dX_1 dX_2 + BT \end{aligned} \tag{S12}$$

where *BT* denotes the boundary terms that can be expressed as

$$BT = \int_{-h}^{0} \left[U_{1,1}\eta - Q\lambda_2\eta - (r+Q)U_{2,2}\eta \right]_0^l dX_2 + \int_0^l \left[U_{1,2}\eta + (r+Q)U_{2,1}\eta \right]_{-h}^0 dX_1 + \int_{-h}^{0} \left[U_{2,1}\zeta + (r+Q)U_{1,2}\zeta \right]_0^l dX_2 + \int_0^l \left[U_{2,2}\eta - Q\lambda_1\varsigma + (r+Q)U_{1,1}\eta \right]_{-h}^0 dX_1 \tag{S13}$$

Equilibrium requires $\delta\Phi$ to be 0 for arbitrary η, ζ and δQ, so we obtain a set of differential equations as from **Supplementary Eqs. S11** and **S12**,

$$\begin{cases} U_{1,11} + U_{1,22} - \left(Q_{,1}U_{2,2} - Q_{,2}U_{2,1}\right) - \lambda_2 Q_{,1} = 0 \\ U_{2,11} + U_{2,22} - \left(Q_{,1}U_{1,2} - Q_{,2}U_{1,1}\right) - \lambda_1 Q_{,2} = 0 \\ \lambda_2 U_{1,1} + \lambda_1 U_{2,2} + U_{1,1}U_{2,2} - U_{1,2}U_{2,1} = 0 \end{cases} \tag{S14}$$

Focusing on a linear perturbation analysis, we further neglect the quadratic terms in **Supplementary Eq. S14** and thus obtain

$$\begin{cases} U_{1,11} + U_{1,22} - \lambda_2 Q_{,1} = 0 \\ U_{2,11} + U_{2,22} - \lambda_1 Q_{,2} = 0 \\ \lambda_2 U_{1,1} + \lambda_1 U_{2,2} = 0 \end{cases} \tag{S15}$$

Supplementary Eq. S15 can be further written as

$$\begin{cases} U_{1,11} + U_{1,22} - r\lambda_1 Q_{,1} = 0 \\ U_{2,11} + U_{2,22} - \lambda_1 Q_{,2} = 0 \\ rU_{1,1} + U_{2,2} = 0 \end{cases} \tag{S16}$$

We are looking for the following periodic solutions

$$\begin{cases} U_1 = Fe^{ksX_2}\sin(kX_1) \\ U_2 = Ge^{ksX_2}\cos(kX_1) \\ \lambda_1 Q = kHe^{ksX_2}\cos(kX_1) \end{cases} \tag{S17}$$

By plugging **Supplementary Eq. S17** in **Supplementary Eq. S16**, we obtain

$$\begin{bmatrix} k^2(s^2-1) & 0 & k^2 r \\ 0 & k^2(s^2-1) & -k^2 s \\ rk & sk & 0 \end{bmatrix}\begin{bmatrix} F \\ G \\ H \end{bmatrix} = 0. \tag{S18}$$

We solve it as

$$\begin{cases} s = r, F = -G, H = Gr^{-1}(r^2-1) \\ s = 1, F = -Gr^{-1}, H = 0 \\ s = -r, F = G, H = -Gr^{-1}(r^2-1) \\ s = -1, F = Gr^{-1}, H = 0 \end{cases}. \tag{S19}$$

Based on the solutions in **Supplementary Eq. S19**, we express the general solution for the incremental displacement as

$$(U_1, U_2) = (\overline{U}_1 \sin kX_1, \overline{U}_2 \cos kX_1), \tag{S20}$$

$$\begin{cases} \overline{U}_1 = -c_1 e^{rkX_2} - c_2 r^{-1} e^{kX_2} + c_3 e^{-rkX_2} + c_4 r^{-1} e^{-kX_2} \\ \overline{U}_2 = c_1 e^{rkX_2} + c_2 e^{kX_2} + c_3 e^{-rkX_2} + c_4 e^{-kX_2} \end{cases} \tag{S21}$$

where c_1, c_2, c_3 and c_4 are undetermined parameters.

We denote N_{ij} as the nominal stress in the *j* direction acting on the face normal to *i* direction. The virtual work associated with $\delta U_{j,i}$ can be expressed as

$$N_{ij}\delta U_{j,i} = N_{11}\delta U_{1,1} + N_{12}\delta U_{2,1} + N_{21}\delta U_{1,2} + N_{22}\delta U_{2,2} \tag{S22}$$

The virtual work can also be expressed as

$$\begin{aligned} \mu\delta I = \mu[U_{1,1} - \lambda_2 Q - (r+Q)U_{1,1}]\delta U_{1,1} + \mu[U_{2,1} + (r+Q)U_{1,2}]\delta U_{2,1} + \\ \mu[U_{1,2} + (r+Q)U_{2,1}]\delta U_{1,2} + \mu[U_{2,2} - \lambda_1 Q - (r+Q)U_{1,1}]\delta U_{2,2} \end{aligned} \tag{S23}$$

By considering $N_{ij}\delta U_{j,i} = \mu\delta I$, we can calculate the nominal stress on the surface as

$$(N_{21}, N_{22}) = (\overline{N}_{21}\sin kX_1, \overline{N}_{22}\cos kX_1) \tag{S24}$$

where

$$\begin{cases} \overline{N}_{21} = -\mu k\left[2c_1 re^{rkX_2} + c_2 r^{-1}\left(r^2+1\right)e^{kX_2} + 2c_3 re^{-rkX_2} + c_4 r^{-1}\left(r^2+1\right)e^{-kX_2}\right] \\ \overline{N}_{22} = -\mu k\left[-c_1 r^{-1}\left(r^2+1\right)e^{rkX_2} - 2c_2 e^{kX_2} + c_3 r^{-1}\left(r^2+1\right)e^{-rkX_2} + 2c_4 e^{-kX_2}\right] \end{cases} \tag{S25}$$

Wrinkling of a neo-Hookean film-substrate structure

Next, we consider a structure consisting of a film with modulus μ_f and thickness H_f bonded on a prestretched semi-infinite substrate with modulus μ_s (**Supplementary Fig. S2b**). The substrate is pre-stretched by $(\lambda_{1s}^0, \lambda_{2s}^0, \lambda_{3s}^0) = (\lambda_p, 1/\lambda_p, 1)$ along X_1, X_2 and X_3 directions, bonded to the film, and then relaxed. Both the film and the substrate are assumed to undergo homogenous deformation at initial state, and the stretches in the film are $(\lambda_{1f}, \lambda_{2f}, 1)$, and in the substrate are $(\lambda_{1s}, \lambda_{2s}, 1) = (\lambda_p\lambda_{1f}, \lambda_{2f}/\lambda_p, 1)$ in X_1, X_2 and X_3 directions. We further denote

$$r_f = r = \lambda_{2f}/\lambda_{1f} \text{ in the film, and} \tag{S26a}$$

$$r_s = \lambda_{2s}/\lambda_{1s} = r/\lambda_p^2 \text{ in the substrate.} \tag{S26b}$$

In the film domain $0 \le X_2 \le H_f$, the traction stress on the free surface should be vanishing, thus

$$(\overline{N}_{21}, \overline{N}_{22}) = 0 \text{ at } X_2 = H_f \tag{S27}$$

From **Supplementary Eqs S27** and S**25**, we have

$$\begin{cases} 2c_1 r e^{rkH_f} + c_2 r^{-1}\left(r^2+1\right)e^{kH_f} + 2c_3 r e^{-rkH_f} + c_4 r^{-1}\left(r^2+1\right)e^{-kH_f} = 0 \\ -c_1 r^{-1}\left(r^2+1\right)e^{rkH_f} - 2c_2 e^{kH_f} + c_3 r^{-1}\left(r^2+1\right)e^{-rkH_f} + 2c_4 e^{-kH_f} = 0 \end{cases} \text{at } X_2 = H_f \tag{S28}$$

On the film-substrate interface $X_2 = 0^{\pm}$, the displacement and surface traction should be continuous, and therefore

$$\frac{1}{\lambda_{1f}\lambda_{3f}}\left(\overline{N}_{21}, \overline{N}_{22}\right)^{+} = \frac{1}{\lambda_{1s}\lambda_{3s}}\left(\overline{N}_{21}, \overline{N}_{22}\right)^{-} \text{ at } X_2 = 0 \tag{S29}$$

where

$$\left(\overline{N}_{21}, \overline{N}_{22}\right)^{+} = \left(2c_1 r + c_2 r^{-1}\left(r^2+1\right) + 2c_3 r + c_4 r^{-1}\left(r^2+1\right), -c_1 r^{-1}\left(r^2+1\right) - 2c_2 + c_3 r^{-1}\left(r^2+1\right) + 2c_4\right) \tag{S30}$$

$$\left(\overline{N}_{21}, \overline{N}_{22}\right)^{-} = \left(2c_1 r + c_2 r_s^{-1}\left(r_s^{2}+1\right) + 2c_3 r_s + c_4 r_s^{-1}\left(r_s^{2}+1\right), -c_1 r_s^{-1}\left(r_s^{2}+1\right) - 2c_2 + c_3 r_s^{-1}\left(r_s^{2}+1\right) + 2c_4\right) \tag{S31}$$

$$\left(\overline{U}_1, \overline{U}_2\right)^{+} = \left(\overline{U}_1, \overline{U}_2\right)^{-}, \text{ at } X_2 = 0 \tag{S32}$$

where

$$\left(\overline{U}_1, \overline{U}_2\right)^{+} = \left(-c_1 - c_2 r^{-1} + c_3 + c_4 r^{-1}, c_1 + c_2 + c_3 + c_4\right) \tag{S33}$$

$$\left(\overline{U}_1, \overline{U}_2\right)^{-} = \left(-c_1 - c_2 r_s^{-1} + c_3 + c_4 r_s^{-1}, c_1 + c_2 + c_3 + c_4\right) \tag{S34}$$

By combining **Supplementary Eqs. S27**, **S29** and S**32**, we obtain the following equation

$$\mathbf{D}\begin{bmatrix} c_1 \\ c_2 \\ c_3 \\ c_4 \end{bmatrix} = \mathbf{0} \tag{S35}$$

The existence of roots requires the determinant of the coefficient matrix $\mathbf{D}$ in **Supplementary Eq. S35** to be zero, *i.e.,* $\det(\mathbf{D})=0$. For wrinkling instability induced by mismatch strain ε_M, the pre-stretch ratio λ_p should be set as a variable as $\lambda_p = 1/\lambda_{1f}$. The resultant critical compressive strain $\varepsilon = 1-\lambda_{1f}$ should be minimized over kH_f. The calculated critical mismatch strain is plotted in **Supplementary Fig. S2C** as a function of μ_f/μ_s.

Parameter estimation of growing/swelling film-substrate systems in reported studies

In **Table 1**, two types of parameters are estimated from the materials used in the films and substrates and marked in gray boxes: $\Gamma/(\mu_s H_f) > 10^3$ indicates the film-substrate adhesion is strong enough to avoid delamination between them; $\mu_f/\mu_s < 10^{-3}$ indicates the modulus of the film is much smaller than that of the substrate. Other parameters are estimated from the data given in the corresponding references and discussed as follow. In reference 5[5], the instability pattern is identified as fold (shown in Fig. 4C in reference 5[5], similar pattern in reference 6[6]). The compression ratio in the film (Endoderm) is g_{fx} =1-0.7, and the compression ratio in the substrate (Mesenchyme) is g_{sx} =0.6-0.5; therefore, the mismatch strain is calculated as $\varepsilon_M = 1-g_{sx}/g_{fx}$ =0.29-0.4. The film-substrate modulus ratio is μ_f/μ_s =1.5-15. In reference 7[7], the analysis is focused on the wrinkling initiation on the brain cortex. The mismatch strain (ε_M =0.05-0.18) is estimated by considering the change of film area, and modulus ratio is larger than 100 and is estimated to be 100-300. In reference 8[8], the differential growth of mucosal region on a softer tissue induces wrinkles in the inner surface of airways. The mismatch strain (ε_M =0.1-0.15) and modulus ratio (μ_f/μ_s =15-50) are given in the paper. Reference 9[9] analyzes wrinkle and period-double on the inner surface of the gut. The mismatch strain is estimated from the growth factors g by using $\varepsilon_M = 1-1/g$, and the modulus ratio (μ_f/μ_s =10-100) is given in the paper. Reference 10[10] is

focused on wrinkling instability of an epithelia cell layer on an elastic stroma. The modulus ratio (μ_f/μ_s ~25) is estimated from the data in the caption of Fig. 1 in reference 10[10]. The mismatch strain is estimated to be 0.05-0.2 from the stability analysis. References 11 and 12[11, 12] demonstrate delamination of the epithelial monolayers on the underlying tissues. The mismatch strain is calculated by using $\varepsilon_M = \varepsilon/(1+\varepsilon)$, where ε is the applied strain in the experiment (11%, 22% and 28%). The shear modulus of the epithelial cell is estimated as 10^4-10^5 Pa [10], and the thickness the epithelial cell layer is estimated as 10 μm. The shear modulus of the silicone substrate is estimated to be in a reasonable range of 10^3-10^5 Pa, and the adhesion energy is estimated as ~10^{-2} J/m^2 ; therefore, the modulus ratio is 1-100 and the normalized adhesion energy $\Gamma/(\mu_s H_f)$ is around 10^{-3}-1. Reference 13[13] investigates the wrinkling instability of blood cell surface. The modulus ratio is directly extracted from the context, and the mismatch strain is estimated by using $\varepsilon_M = \varepsilon/(1+\varepsilon)$, where ε is the growth strain. Reference 14[14] studies the delaminated-buckles on growing biofilm on a culture gel. The shear modulus of the biofilm is μ_f =2.7-16.5 kPa, and the shear modulus of the agar gel substrate is estimated as μ_s =1-100 kPa; so the modulus ratio is around μ_f/μ_s =0.3-16. The thickness of the biofilm is H_f =0.5-1 mm; the adhesion energy density is estimated as $\Gamma = 0.01 - 0.1 J/m^2$ [15]; the normalized adhesion energy is thus calculated as $\Gamma/(\mu_s H_f) \approx 10^{-3}$ -0.2. The mismatch strain 0-0.4 is estimated from the cross-section images of the buckled biofilm. The delaminated buckle pattern of the biofilm in reference 14[14] is very similar to the patterns in references 16 and 17[16, 17]. References 18 and 19[18, 19] are focused on wrinkling instability on fruits and plants. The mismatch strain is directly extracted from the relative magnitude of shrinkage, and modulus ratio is from the context. When the mismatch strain is larger than 0.3, the wrinkle mode transits into a period-double-like mode. A similar wrinkling instability of a drying apple is analyzed in reference 20[20]. References 21-24[21-24] analyze creasing instability in a swelling hydrogel on a rigid substrate. The critical mismatch strain for the creasing instability is observed as 0.32-0.38 [22, 23]. In reference 25[25], crease,

wrinkle and fold have been observed in bilayer swelling hydrogel system. If $\mu_f/\mu_s < 1$, creases emerge on the outer ring of the hydrogel; however, if $\mu_f/\mu_s > 1$, the system first wrinkles and then transits into the fold mode. The corresponding shear modulus and mismatch strain are directly extracted from the context of reference 25[25]. The wrinkle to fold transition has also been observed in reference 26[26], and the experimental data are directed extracted from its context. Reference 27[27] is focused on creasing to delaminated-buckling in a swollen silicone film (Sylgard, base:cross-linker=10:0.4) bonded on a rigid substrate. The swelling ratio of Sylgard in toluene by weight is 380%; the density of toluene and Sylgard are 866.90 kg/m^3 and 965.00 kg/m^3, respectively; the swelling ratio by volume is thus estimated as 412%; the maximum mismatch strain is then calculated as $\varepsilon_M \approx 0.51$. The film thickness is $H_f \approx 20$μm, and its shear modulus is μ_f=18 kPa. The shear modulus of acrylic substrate is ~100 MPa. The film-substrate adhesion is estimated as 0.1-10 J/m^2, so the normalized adhesion energy is estimated as $\Gamma/(\mu_s H_f) \approx$ 5×10^{-5}-5×10^{-3}. Reference 28[28] studies wrinkling instability of a swelling UV-ozone treated Sylgard thin film on a normal Sylgard. The mismatch strain is estimated by the swelling ratio. The shear moduli of the UV-ozone treated film and the Sylgard substrate are estimated as ~1 GPa and 100 kPa, respectively; so the modulus ratio is around 10^4.

Supplementary References

1. Cao, Y., Hutchinson, J. W. From wrinkles to creases in elastomers: the instability and imperfection-sensitivity of wrinkling. *Proc. R. Soc. A* **468**, 94-115 (2012).

2. Cao, Y., Hutchinson, J. W. Wrinkling phenomena in neo-Hookean film/substrate bilayers. *J. Appl. Mech.* **79**, 1019 (2012).

3. Hutchinson, J. W. The role of nonlinear substrate elasticity in the wrinkling of thin films. *Phil. Trans. R. Soc. A* **371**, (2013).

4. van der Heijden, A. M. *WT Koiter's elastic stability of solids and structures* (Cambridge University Press Cambridge, 2009).

5. Shyer, A. E., *et al.* Villification: How the Gut Gets Its Villi. *Science* **342**, 212-218 (2013).

6. Amar, M. B., Jia, F. Anisotropic growth shapes intestinal tissues during embryogenesis. *Proc. Natl. Acad. Sci. U.S.A.* **110**, 10525-10530 (2013).

7. Richman, D. P., Stewart, R. M., Hutchinson, J. W., Caviness Jr, V. S. Mechanical model of brain convolutional development. *Science* **189**, 18-21 (1975).

8. Wiggs, B. R., Hrousis, C. A., Drazen, J. M., Kamm, R. D. On the mechanism of mucosal folding in normal and asthmatic airways. *J. Appl. Physiol.* **83**, 1814-1821 (1997).

9. Wang, Q., Tahir, M., Zhang, L., Zhao, X. Electro-creasing instability in deformed polymers: experiment and theory. *Soft Matter* **7**, 6583-6589 (2011).

10. Hannezo, E., Prost, J., Joanny, J. F. Instabilities of Monolayered Epithelia: Shape and Structure of Villi and Crypts. *Phys. Rev. Lett.* **107**, 078104 (2011).

11. Eisenhoffer, G. T., *et al.* Crowding induces live cell extrusion to maintain homeostatic cell numbers in epithelia. *Nature* **484**, 546-549 (2012).

12. Marinari, E., *et al.* Live-cell delamination counterbalances epithelial growth to limit tissue overcrowding. *Nature* **484**, 542-545 (2012).

13. Wang, L., Castro, C. E., Boyce, M. C. Growth strain-induced wrinkled membrane morphology of white blood cells. *Soft Matter* **7**, 11319-11324 (2011).

14. Asally, M., *et al.* Localized cell death focuses mechanical forces during 3D patterning in a biofilm. *Proc. Natl. Acad. Sci. U.S.A.* **109**, 18891-18896 (2012).

15. Chen, M., Zhang, Z., Bott, T. Direct measurement of the adhesive strength of biofilms in pipes by micromanipulation. *Biotechnol. Tech.* **12**, 875-880 (1998).

16. DePas, W. H., *et al.* Iron induces bimodal population development by Escherichia coli. *Proc. Natl. Acad. Sci. U.S.A.* **110**, 2629-2634 (2013).

17. Wilking, J. N., *et al.* Liquid transport facilitated by channels in Bacillus subtilis biofilms. *Proc. Natl. Acad. Sci. U.S.A.* **110**, 848-852 (2012).

18. Li, B., *et al.* Surface wrinkling patterns on a core-shell soft sphere. *Phys. Rev. Lett.* **106**, 234301 (2011).

19. Yin, J., *et al.* Stress-driven buckling patterns in spheroidal core/shell structures. *Proc. Natl. Acad. Sci. U.S.A.* **105**, 19132-19135 (2008).

20. Cerda, E., Mahadevan, L. Geometry and physics of wrinkling. *Phys. Rev. Lett.* **90**, 074302 (2003).

21. Kim, J., Yoon, J., Hayward, R. C. Dynamic display of biomolecular patterns through an elastic creasing instability of stimuli-responsive hydrogels. *Nat. Mater.* **9**, 159-164 (2010).

22. Trujillo, V., Kim, J., Hayward, R. C. Creasing instability of surface-attached hydrogels. *Soft Matter* **4**, 564-569 (2008).

23. Yoon, J., Kim, J., Hayward, R. C. Nucleation, growth, and hysteresis of surface creases on swelled polymer gels. *Soft Matter* **6**, 5807-5816 (2010).

24. Tanaka, T., *et al.* Mechanical instability of gels at the phase transition. *Nature* **325**, 796-798 (1987).

25. Dervaux, J., Couder, Y., Guedeau-Boudeville, M.-A., Amar, M. B. Shape transition in artificial tumors: from smooth buckles to singular creases. *Phys. Rev. Lett.* **107**, 018103 (2011).

26. Sultan, E., Boudaoud, A. The Buckling of a Swollen Thin Gel Layer Bound to a Compliant Substrate. *J. Appl. Mech.* **75**, 051002-051002 (2008).

27. Velankar, S. S., Lai, V., Vaia, R. A. Swelling-Induced Delamination Causes Folding of Surface-Tethered Polymer Gels. *ACS Appl. Mater. Interfaces* **4**, 24-29 (2012).

28. Breid, D., Crosby, A. J. Effect of stress state on wrinkle morphology. *Soft Matter* **7**, 4490-4496 (2011).

29. Jiang, H., *et al.* Finite deformation mechanics in buckled thin films on compliant supports. *Proceedings of the National Academy of Sciences* **104**, 15607-15612 (2007).

30. Khang, D. Y., Jiang, H., Huang, Y., Rogers, J. A. A stretchable form of single-crystal silicon for high-performance electronics on rubber substrates. *Science* **311**, 208-212 (2006).
